# Characterization of two keystone taxa, sulfur-oxidizing, and nitrate-reducing bacteria, by tracking their role transitions in the benzo[a]pyrene degradative microbiome

**DOI:** 10.1186/s40168-023-01583-1

**Published:** 2023-06-24

**Authors:** Xiaolan Lin, Baoyi Qiao, Ruirui Chang, Yixin Li, Wei Zheng, Zhili He, Yun Tian

**Affiliations:** 1 Key Laboratory of the Ministry of Education for Coastal and Wetland Ecosystems, School of Life Sciences, Xiamen University, Xiamen, 361102 China; 2 State Key Laboratory of Marine Environmental Science, Xiamen University, Xiamen, 361102 China; 3 Southern Marine Science and Engineering Guangdong Laboratory (Zhuhai), Zhuhai, 519080 China

**Keywords:** Co-occurrence network, Keystone taxa, Sulfur-oxidizing and nitrate-reducing bacteria, Metagenome-assembled genomes, BaP degradative microbiome

## Abstract

**Background:**

Keystone taxa are drivers of microbiome structure and functioning, which may play critical roles in microbiome-level responses to recalcitrant pollution and are a key to bioremediation. However, the characterization and manipulation of such taxa is a major challenge due to the complexity of microbial communities and rapid turnover in both time and space. Here, microcosms were set up with benzo[a]-pyrene (BaP) and/or nitrate based on C-rich, S-rich, and N-limited mangrove sediments as reductive experimental models to trigger and track the turnover of keystone taxa to address this challenge.

**Results:**

Based on microbial co-occurrence network analysis, two keystone taxa, *Sulfurovum* and *Sulfurimonas*, were found to exhibit significant role transitions in different microcosms, where these two taxa played nonkeystone roles with neutral relationships in in situ mangrove sediments. However, *Sulfurimonas* transitioned to be keystone taxa in nitrate-replenished microcosms and formed a keystone guild with *Thioalkalispira*. *Sulfurovum* stood out in BaP-added microcosms and mutualized in a densely polycyclic aromatic hydrocarbon (PAH)-degrader-centric keystone guild with *Novosphingobium* and *Robiginitalea*, where 63.25% of added BaP was removed. Under the occurrence of nitrate and BaP, they simultaneously played roles as keystone taxa in their respective guilds but exhibited significant competition. Comparative genomics and metagenome-assembled genome (MAG) analysis was then performed to reveal the metabolic potential of those keystone taxa and to empirically deduce their functional role in keystone guilds. *Sulfurimonas* possesses a better sense system and motility, indicative of its aggressive role in nitrate acquisition and conversion; *Sulfurovum* exhibited a better ability for oxidation resistance and transporting nutrients and electrons. High-efficiency thermal asymmetric interlaced polymerase reaction (hiTAIL-PCR) and enhanced green fluorescent protein (eGFP)-labeling approaches were employed to capture and label the BaP key degrader to further experimentally verify the roles of keystone taxa *Sulfurovum* in the keystone guilds. Observations of the enhancement in reactive oxygen species (ROS) removal, cell growth, and degradation efficiency by co-culture of isolated keystone taxa strains experimentally demonstrated that *Sulfurovum* contributes to the BaP degradative microbiome against BaP toxicity.

**Conclusions:**

Our findings suggest that the combined use of co-occurrence network analysis, comparative genomics, and co-culture of captured keystone taxa (3C-strategy) in microbial communities whose structure is strongly shaped by changing environmental factors can characterize keystone taxa roles in keystone guilds and may provide targets for manipulation to improve the function of the microbiome.

Video Abstract

**Supplementary Information:**

The online version contains supplementary material available at 10.1186/s40168-023-01583-1.

## Background

Microbiomes are key components across diverse ecosystems, and they have been proven to have a critical impact on the health and function of hosts and surrounding environments. Pioneering studies have attempted to exploit their functional features and selectively inoculate them into hosts to improve host health, resistance to adverse conditions, and agricultural productivity and even determine solutions for human disease [1, 2]. These studies demonstrated that targeted probiotic treatments could be an effective and promising therapy to tweak host health, and the selection of functional microorganisms is fundamental to successful treatments. Similarly, environments suffering from contamination could be regarded as “patients,” and there remains an urgent need for microbial therapies to remediate pollution, which is important for environmental restoration and human health. To date, a series of functional microorganisms with degrading capability has been continuously identified and isolated. However, different pollutants exhibit different levels of toxicity and persistence in environments, which could affect their biodegradability to a great extent. Unlike pollutants with simpler structures, such as oxynitride and alkanes, which can be degraded efficiently [3, 4], remediating environments contaminated by highly toxic and persistent pollutants is challenging. Among these hard-treated pollutants, high molecular weight polycyclic aromatic hydrocarbons (HMW-PAHs) are important components of petroleum products and are widely distributed. These compounds are composed of four or more fused benzene and/or pentacyclic rings, making them highly hydrophobic and recalcitrant. One such HMW-PAH is benzo[a]-pyrene (BaP), a five-ring compound that is reported to cause genotoxic effects and even carcinogenicity to a broad range of prokaryotes and mammals; thus, its occurrence and fate of this compound in the environment are of great concern [5]. To date, the biodegradation of HMW-PAHs remains a difficult issue to conquer due to their high biotoxicity, and efficient microbial degraders have been discovered to be scarce. HMW-PAH degradation in natural environments is always a microbiome-level function that involves diverse microorganisms in cooperative metabolic routes [6], suggesting that recreating this cooperation in artificial degrading consortia would be one possible avenue for HMW-PAH degradation. Thus, identifying and manipulating ideal targets from complex microbiota would be an essential issue, not only for “therapy” to eliminate HMW-PAH pollution but also for other similar cases that suffer from a lack of key players. However, it is still a challenging task, considering the inestimable number of microorganisms and the complexity of their metabolic features and interactions with biotic and abiotic environments.

The discovery of keystone taxa from ecosystemic communities provides an operable solution. The concept of keystone taxa has been transferred from macroecology to microecology [7] and defined as those microorganisms that exert considerable influence on the microbial community, and their removal has been computationally shown to cause a drastic shift in microbiome structure and performance [8]. As an increasing number of keystone taxa have been identified across diverse environments from the human gut and plant rhizosphere to oil-contaminated sites, an increasing number of studies have explored the ecological importance of keystone taxa and inferred that (1) keystone taxa can orchestrate microbial communities to perform ecosystem functions, so they are referred to as “ecosystem engineers” or drivers of microbiome structure and functioning [9] and (2) keystone taxa are strongly driven by environmental change and even show higher sensitivity than other taxa [10], so keystone taxa can also be regarded as good indicators of particular environmental factors. However, unlike the keystone taxa that can be removed from the community to assess their role in macroecology, the identification, characterization, and manipulation of such taxa are still a tough challenge in microecology due to the high complexity of the microbiome and inherent interactions, and adequate methods are needed to complement their characterization.

Many studies have identified potential keystone taxa using co-occurrence network analysis in terms of their topological importance. Using this approach, several studies identified keystone taxa in contaminated sites, such as *Nitrospira* in oil-contaminated soils [11], *Pseudomonas* in a rare earth element mine [12], Planctomycetanceae in heavy-metal-contaminated soils [13], and *Rhodobacter* in response to water pollution [14]. This computational approach is powerful but may lead to inevitable computational bias and equivocal results that cannot be used to assess their importance to the microbiome just from a snapshot in a particular situation since their functionality is commonly context-dependent [9]. Previous studies have indicated that keystone species change with environmental changes and only play roles under certain conditions, suggesting that different keystone taxa are strongly selected by different environmental factors and play different roles under different situations [15, 16]. Therefore, facing the difficulty of determining the ideal targets to manipulate for contaminated environments, keystone taxa provide an opportunity considering their decisive influence on microbiome structure and functioning. Their identification and characterization are crucial for further manipulation but are still challenging tasks. PAH-degrading microbial communities on PAH-contaminated theaters are, however, an attractive setting to start testing systems biology-derived models and hypotheses, as they are relatively simple in diversity and key activities, with several key players being isolated and a high availability of experimental data and approaches. Therefore, biodegradation of PAHs provides a suitable and realistic starting point to achieve our goals and to pose and test the hypotheses that what and how microbial keystone taxa individually or in a guild exert a considerable influence on microbiome structure and functioning in PAH-contaminated theaters. In the present study, mangrove sediments were used to set up microcosms treated with BaP contamination and/or nitrate addition as environmental factor changes, from which eligible 16S rRNA gene amplicons and metagenomes were obtained. These datasets were then applied to cooccurrence network analysis, comparative genomics, and metagenome assembled genome (MAG) analysis to identify the keystone taxa, trace their polymicrobial interactional dynamics under BaP contamination and nitrate biostimulation, and address their metabolic potential, in combination with the experimental test of co-culture of isolated key players inferring their keystone significance to the PAH-degrading microbiome and providing valuable clues for characterization and manipulation of microbial keystone taxa.

## Methods

### Sample collection and establishment of the microcosm

Sample collection was performed according to our previous study [17]. The mangrove sediment microcosms were built in transparent cylindrical aquaria (diameter = 15 cm, height = 45 cm for each). These aquaria were soaked beforehand with 2 M HCl overnight and washed with distilled water. To protect the PAHs from photodegradation, the microcosms were packed with aluminum foil. For BaP treatment, BaP (2.4 g) was first dissolved in an appropriate volume of dichloromethane and then intensively mixed with 360 g of dry sediment powder in round-bottomed flasks. Finally, a mixture of BaP with dry sediment was obtained by thoroughly volatilizing dichloromethane using a vacuum rotary evaporator. The mixtures were stored in the dark before use.

Before setting up microcosms, the sediments were thoroughly mixed and 9 samples were collected randomly to present samples in day 1. Each microcosm received approximately 4 kg of mangrove sediment. Then, the prepared BaP mixtures were added to the microcosms and fully mixed to establish BaP-contaminated microcosms (named B) with initial contamination levels of 200 ppm BaP. In the treatments with nitrate addition, 100 ml of 150 mM NaNO_3_ solution was added and thoroughly mixed to yield an initial nitrate concentration of 310 mg/kg (named N). Meanwhile, we constructed microcosms with both BaP and nitrate (as BN) added, along with the control microcosms (as CK). Triplicate microcosms were established for each treatment. These 12 microcosms were incubated at ambient temperature (between 20 and 25 °C) for 90 days. During incubation, distilled water was added to each microcosm to replace the evaporated water. Triplicate samples were collected from each microcosm at different time points: 30, 60, and 90 days. Overall, 117 samples were collected, representing samples of B, N, BN, and CK microcosms.

### Physicochemical assays and BaP analysis in microcosms

For quantitative measurement of nitrate and ammonium concentrations aiming at tracing nitrate utilization, 3 g sediments were collected for each sample and mixed with 15 mL KCl solution (2 mM). After incubation at 30 °C for 2 h, the mixture was centrifuged at 6000 rpm for 5 min, and the supernatant was then filtered with a 0.45-μm membrane. Then, the supernatant was used for the measurement of nitrate and ammonium concentrations via the AA3 platform (Bran-Luebbe, Germany) and indophenol blue photometric method, respectively.

To track the BaP concentration during the incubation time, 30 g of sediment was taken from each sample and freeze-dried. Then, the samples were passed through an 80-mesh screen sieve, and 20 g sediments were transferred to a 250 mL prewashed conical flask. The sediments were spiked with 20 μL internal standard prior to extraction, shaken with 100 mL dichloromethane, and sonicated in an ultrasonic bath (50 °C) for 20 min. After the extracts were transferred, a second extraction was conducted with another 100 mL dichloromethane. The two extracts were then combined and purified with double distilled water. The purified products were concentrated by rotary evaporation to 1 mL. The solvent extracts were then filtered with a 0.22-μm membrane and subjected to high-performance liquid chromatography (HPLC) to assay the BaP concentration.

### 16S rRNA gene sequencing and statistical analysis

These 117 samples were subjected to 16S rRNA gene sequencing. The sequencing and sequence processing methods were described in our previous study [17], and an operational taxonomic unit (OTU) table was generated. We performed calculation of α-diversity indices, principal component analysis (PCA) on the relative abundance of taxonomic profiles, and Adonis test (the number of permutations was set as 999) and dispersion analysis using ieggr and vegan packages in the R software platform (http://www.r-project.org/).

### Network construction and keystone taxa identification

Phylogenetic molecular ecological networks (pMENs) under four treatments were constructed using the Molecular Ecological Network Analysis Pipeline [18] based on the OTU table, which is open-accessible on the website (http://ieg2.ou.edu/MENA). Time-series pMENs (TMEN) of four treatments were constructed across all time points (36 samples for each network), with the time lagging option chosen, and these four networks were regarded as B-TMEN (BaP contamination), N-TMEN (nitrate addition), BN-TMEN (BaP and nitrate contamination), and CK-TMEN (no addition). The finest similarity threshold (*St*) to define the connections among nodes was automatically chosen by the random matrix theory (RMT) method in the pipeline. Meanwhile, the same datasets were applied to the Sparse Correlations for Compositional data (SparCC) method to construct another four networks named B-SN (BaP contamination), N-SN (nitrate addition), BN-SN (BaP and nitrate contamination), and CK-SN (no addition) (correlation value ≥ 0.6). The credibility of network connections was controlled by 100 × permutation-based false-positive rate calculation (*p* < 0.05). These SparCC networks were generated within R using the SPIEC-EASI package [19]. After network construction, subsequent network visualization and editing were realized by Cytoscape v3.3.0 [20].

In this study, the keystone taxa were characterized by their topological roles in microbial networks. The topological role of each node was determined based on within-module connectivity (*Zi*) and among-module connectivity (*Pi*). When a node had *Zi* > 2.5 or/and *Pi* > 0.62, it was regarded as a keystone taxon [18].

### Comparative genomics of selected keystone taxa

Based on the comparative network analysis, two keystone taxa, *Sulfurovum* and *Sulfurimonas*, were found to exhibit significant role transitions in different microcosms and then were selected for comparative genomic analysis. A total of 14 and 12 high-quality representative genome sequences of *Sulfurimonas* and *Sulfurovum* were collected from the EzBioCloud database [21], including complete genomes sequenced from pure culture or assemblies from metagenomic binning.

#### Phylogenomic analysis

Phylogenomic analysis was conducted using a core genome alignment concatenation approach. Based on a set of 49 core universal genes defined by clusters of orthologous groups (COGs) gene families extracted from genomes, a phylogenetic tree for the set of 30 genomes from the two species was built and visualized using FastTree2 and MEGA7 [22].

#### Pangenomic analysis

Pangenomic analysis was performed based on the *Sulfurimonas* and *Sulfurovum* genomes. After all, the protein sequences in a genus were compared using a basic local alignment search tool P (BLASTP) all-against-all search with an *E* value cutoff of < 1e − 05 and percent match length ≥ 50%, orthologous groups were delimited using OrthoMCL [23]. The putative pangenome, core genome, and lineage-specific gene sets were extracted from the OrthoMCL output, where a pangenome was the set of protein-coding genes in all the selected organisms, including genes present in all organisms (core genome) and genes present only in some organisms. Finally, pangenomes of *Sulfurimonas* and *Sulfurovum* were obtained.

#### Functional annotation

Gene annotations of representative genomes were performed on Prokka v1.11 [24], and the functional categories were classified against Kyoto Encyclopedia of Genes and Genomes (KEGG) and Gene Ontology (GO) terms by a combination of DIAMOND and MEGAN v6.6.5. To obtain the transporter profile of *Sulfurovum*, the pangenome of *Sulfurovum* was blasted against transport classification database (TCDB) [25]. To scan whether *Sulfurovum* is capable of extracellular electron transfer, the pangenome of *Sulfurovum* was blasted against a database curated from the Swiss-Prot and TrEMBL databases [26].

#### Statistical analyses

The functional categories (KEGG pathways and GO terms at multiple levels) with significantly different abundances of *Sulfuimonas* and *Sulfurovum* genomes were identified using analysis of variance (ANOVA) with Benjamini–Hochberg false discovery rate (FDR) correction performed on statistical analysis of taxonomic and functional profiles (STAMP) v2.1.3 [27].

Heatmaps and bubble charts were generated within the R software platform using the ggplot2 (http://had.co.nz/ggplot2/) package.

### Metagenome sequencing and metagenome-assembled genome (MAG) retrieval

Samples from day 1 and day 60 were chosen for shotgun sequencing. The metagenomic sequencing and sequence processing methods were described in our previous study [17]. After assembly, contigs of each sample whose length was longer than 1000 bp were applied to bin MAGs using MetaBAT2 [28] and MaxBin2 [29]. The taxonomic assignments of MAGs were obtained based on the Genome Taxonomy Database (GTDB) v1.1.0 [30]. The MAGs were refined with RefineM [31] and the Anvio pipeline [32]. Then, the quality of the refined MAGs was checked using CheckM [33]. The *Sulfurimonas* and *Sulfurovum* MAGs with moderate quality remained to perform the gene annotation using Prokka v1.13.7. The annotated protein sequences were then applied to BLASTKOALA [34] to obtain their functional categories and reconstruct their metabolic pathways, which were compared to those of *Sulfurimonas*/*Sulfurovum* representative genomes.

### Capture and eGFP labeling of the BaP key degrader Novosphingobium strain

#### High-efficiency thermal asymmetric interlaced PCR (hiTAIL-PCR)

OTU5484 was identified as a keystone taxon under BaP contamination affiliated with *Novosphingobium*, which has been reported as a PAH degrader. To obtain precise taxonomic information on OTU5484 at the species/strain level, degenerate primer sets for OTU5484 were designed based on their sequencing reads retrieved from 16S rRNA gene datasets. The degenerate primer sets were designed using CODEHOP and evaluated using fastPCR. Then, the primer sets were applied to the hiTAIL-PCR experiment to obtain complete 16S rRNA gene sequences of OTU5484, followed by taxonomic assignment against the nonredundant (NR) database using the BLAST method. According to the results, the closest related species, *N. pentaromativorans* US6-1, was selected for subsequent co-culture as a representative strain of OTU5484.

#### Construction of eGFP-labeled N. pentaromativorans US6-1

To detect the growth of *N. pentaromativorans* US6-1 in co-culture systems in real time, eGFP-labeled *N. pentaromativorans* US6-1 was constructed. *Escherichia coli* DH5α was precultured in LB liquid medium and incubated at 37 °C until the final cell concentration reached OD_600nm_ = 0.3, followed by centrifugation (4 °C, 6000 rpm) for 8 min to harvest the cells. Then, the cells were added to 30 mL CaCl_2_-MgCl_2_ solution (CaCl_2_: 20 mM, MgCl_2_: 80 mM) and incubated for 40 min on ice, followed by centrifugation (4 °C, 4100 rpm) for 10 min. After the supernatant was discarded, 2 mL CaCl_2_ (100 mM) and 70 μL dimethyl sulfoxide (DMSO) were added and incubated on ice for 15 min. Another 70 μL of DMSO was added to the tube and incubated on ice for 15 min. Then, these competent cells were stored in a − 80 °C refrigerator.

The peGFP plasmids of *E. coli* DH5α were extracted with a high-purity plasmid preparation kit (Hlingene, China), and the eGFP gene fragment was amplified using the primers eGFP-F-BamH I (GGATCCCCATGGTGAGCAAGGGCGA) and eGFP-R-EcoR I (GAATTCC TTACTTGTACAGCTCGTC) before adding two enzyme cleavage sites, BamH I and EcoR I. Restriction enzyme digestion was then performed on the eGFP fragment and plasmid pRK415 simultaneously using BamH I and EcoR I. Treated fragments and plasmids were ligated to form recombinant plasmids. These recombinant plasmids were transferred into *E. coli* DH5α using the heat shock method.

For the introduction of peGFP plasmids, *E. coli* HB101 was used as helper strain to provide cytoplasmic bridge from donor bacterium DH5α to recipient strain US6-1. The donor bacterium DH5α and the helper bacterium HB101 were precultured in LB liquid medium containing tetracycline and kanamycin at 37 °C for 12 h, when the recipient bacterium US6-1 was cultured in 2216E liquid medium at 30 °C for 36 h, followed by centrifugation at 6000 rpm for 5 min, and the cells were washed three times with 10 mM MgSO_4_ solution. After mixing these cell resuspensions in the ratio of donor bacteria:auxotrophic bacteria:recipient bacteria = 1:1:2, the mixture was centrifuged at 6000 rpm for 8 min, resuspended with 100 μL of MgSO_4_ solution, and then added dropwise to 2216E solid medium before incubating at 30 °C for 24 h. The colonies on the plates were eluted and diluted 10 times, 50 times, and 100 times using 10 mM MgSO_4_ solution. Then, 100 μL of the dilution was applied to a 2216E plate with 50 μg/mL cefadroxil and 34 μg/mL tetracycline and incubated at 30 °C in the dark. Then, orange single colonies were picked for PCR experiments to verify whether they were transformants.

#### Co-culture of key PAH-degrading strain with Sulfurimonas and Sulfurovum strains

The co-culture systems of US6-1 with *Sulfurimonas*/*Sulfurovum* were launched to experimentally verify their interactional pattern. Considering that few isolated strains of *Sulfurovum* and *Sulfurimonas* have been obtained to date, after checking genome-informed pathways, two type strains of *Sulfurovum* and *Sulfurimonas* named *Sulfurovum indicum* ST-419^ T^ (= MCCC1A17954^T^) and *Sulfurimonas indica* NW8N^T^ (= MCCC1A13988^T^) were selected for the co-culture experiments. Wild-type US6-1 and eGFP-labeled US6-1 (eGFP-US6-1) were precultivated in 2216E medium at 30 °C, while *Sulfurovum indicum* and *Sulfurimonas indica* were precultivated in MMJHS medium [35] at 37 °C until the late log phase of growth. The cells were collected by centrifugation (6000 rpm for 20 min), washed three times with MM2 medium, and suspended in MM2 medium.

To track the growth of US6-1, eGFP-labeled US6-1 cell suspension was used to co-culture with *Sulfurovum indicum* and *Sulfurimonas indica* cell suspensions in 20 mL MM2 medium containing 10 ppm BaP with starting OD_600nm_ 0.3, regarded as cell-based co-culture (eGFP). Mono-cultures (eGFP-US6-1) were constructed under the same conditions without *Sulfurovum indicum* and *Sulfurimonas indica* cells to be used as controls. The experiments were performed in triplicate. The samples were collected each day from day 1 to day 11, and their fluorescence intensity was measured by ELIASA and converted into the biomass of US6-1.

Meanwhile, the spent culture supernatants of *Sulfurovum indicum* and *Sulfurimonas indica* with different starting inoculum conditions (10% and 20% inoculum sizes in MMJHS medium and 10% inoculum size under stress of 10 ppm BaP in MM2) were retained after centrifugation, followed by filter sterilization (0.22 μm). Then, wild-type US6-1 cells were transferred into 20 mL supernatants containing 10 ppm BaP at OD_600nm_ 0.3 to construct supernatant-based co-cultures. The mono-cultures (only US6-1) were constructed in the same manner when the supernatants were replaced with MM2 medium to be used as controls. The experiments were performed in triplicate. Then, the reactive oxygen species (ROS) levels of all the cultures at 0 h, 2 h, 6 h, 12 h, and 24 h were determined using a reactive oxygen species assay kit (Beyotime, Jiangsu, China).

**Fig. 1:**
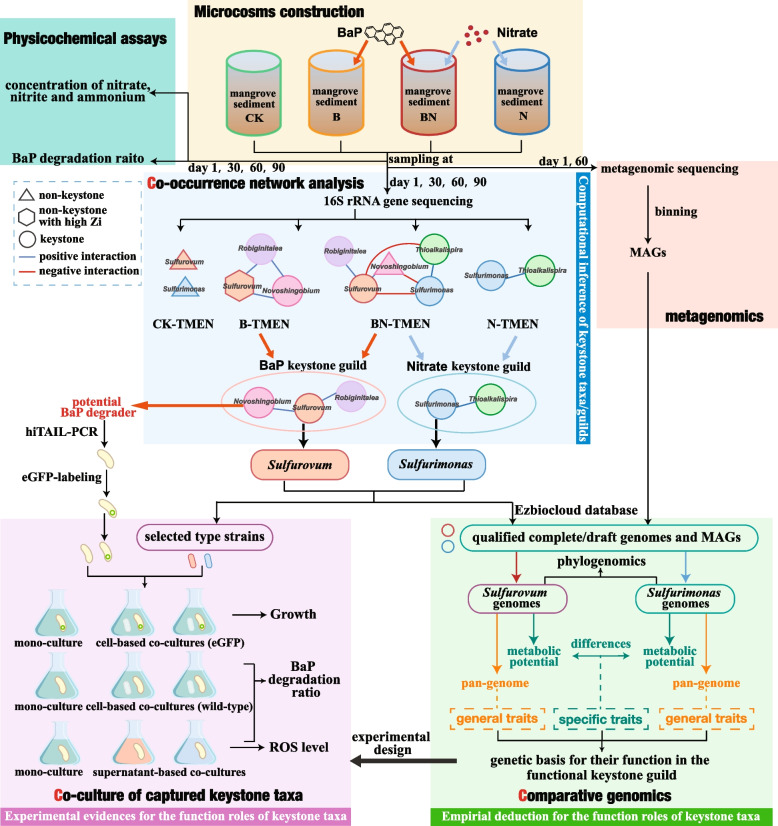
Experimental design and combining computational pipeline (3C-strategy)

Simultaneously, the co-cultures of wild-type US6-1 with *Sulfurovum indicum/Sulfurimonas indica* were also constructed in the same manner as cell-based co-cultures (eGFP) and regarded as cell-based co-cultures (wild-type). The control samples were prepared with 20 mL of MM2 medium containing 10 ppm BaP. Subsequently, the samples were collected from the mono-cultures (wild-type US6-1), cell-based co-cultures (wild-type), supernatant-based co-cultures, and controls at days 1, 2, 4, and 6 to measure the concentrations of BaP [36]. All experiments were conducted in triplicate. The experimental design and combined computational pipeline are illustrated in Fig. 1.

## Results

### Nitrate transformation and BaP degradation rates in microcosms

**Fig. 2:**
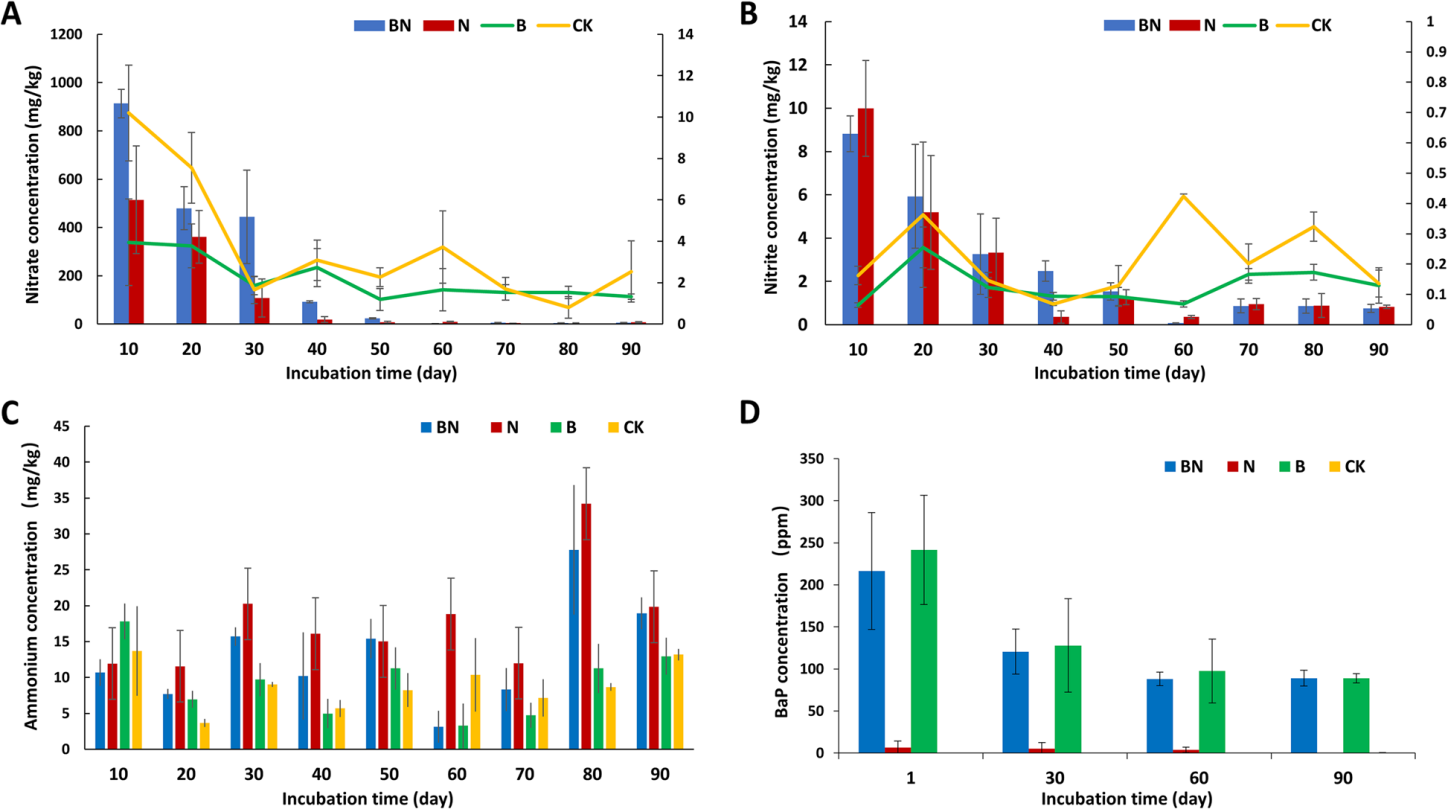
Nitrate transformation and BaP degradation rates in microcosms. The nitrate concentration (**A**), nitrite concentration (**B**), ammonium concentration (**C**), and BaP degradation ratios (**D**) in the microcosms. BN, N, B, and CK indicate the microcosms under BaP and nitrate addition, nitrate replenishment, BaP contamination, and without any addition

To reveal the capacity of the mangrove microbiome for BaP degradation and whether the addition of nitrate to nitrate-limited sediments facilitates BaP degradation, the degradation ratios of BaP and nitrate were measured (Fig. 2). Nitrate was transformed significantly after 30 days and was not detected after 60 days in the BN and N microcosms, when the concentration of ammonium tended to be higher in the nitrate-replenished samples (3.1–34.2 ppm) than in the others (3.3–17.8 ppm) after 70 days, indicative of nitrate utilization by the mangrove microbiota. Regarding the BaP degradation ratios, significant degradation occurred after 30 days, and the degradation ratios reached 58.90% and 63.25% in the BN and B microcosms.

### The diversity of mangrove microbial communities under different treatments

**Fig. 3:**
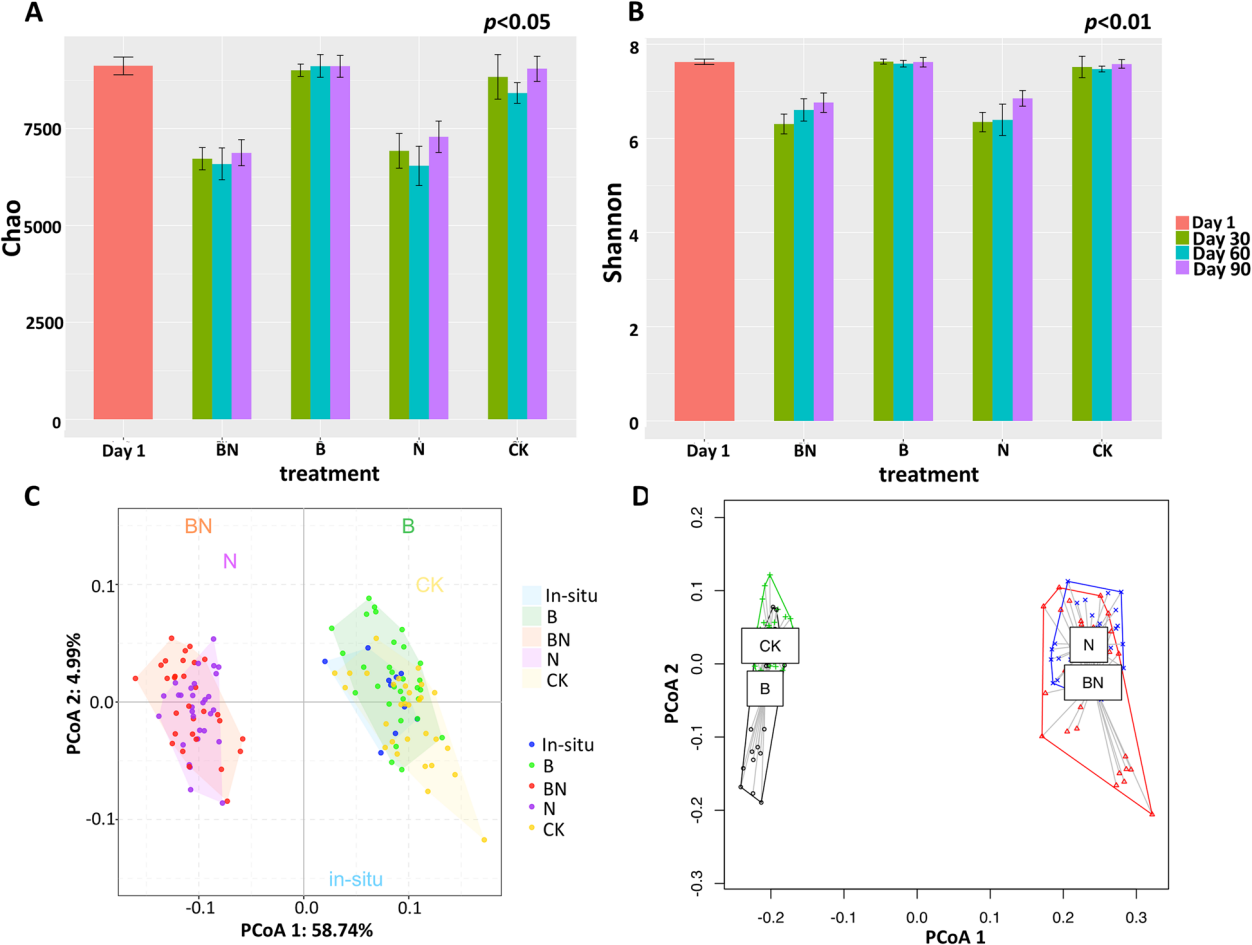
The effects of BaP contamination and nitrate replenishment on microbial diversity and community succession. The α-diversity index values of microbial communities in microcosms, including **A** Chao and **B** Shannon. **C** The plot of PCA. **D** The dispersion degree of microbial community structures under different treatments during 90-day incubation. BN, N, B, and CK indicate the microcosms under BaP and nitrate addition, nitrate replenishment, BaP contamination, and without any addition

After sequencing and read-processing, 33,097 high-quality reads were obtained per sample and then clustered into OTUs in the range of 3424–6275 per sample. The α-diversity index of samples under nitrate treatment (BN and N) decreased significantly (*p* < 0.05), when Chao decreased from 9118.7 ± 225.3 in day 1 to 6723.7 ± 285.3 (BN) and 6929.6 ± 451.0 (N) after 30 days, Shannon value decreased from 7.6 ± 0.057 to 6.3 ± 0.21 (BN) and 6.4 ± 0.21 (N). Meanwhile, the Chao and Shannon values of samples from the B and CK microcosms remained stable, ranging from 9007.2 ± 158.4 to 9115.3 ± 294.2 for the Chao index and 0.0015 ± 0.00010 to 0.0016 ± 0.00029 for the Shannon index (Fig. 3). In addition, the phylogenetic α-diversity indices, including Faith’s PD, Rao’s entropy, and mean phylogenetic distance (MPD), displayed similar trends (Additional file 1: Fig. S1).

Regarding β-diversity, PCoA illustrated that the microbial community structures of samples under nitrate biostimulation (BN and N microcosms) were obviously clustered together and separated from the others, while a less obvious community succession was also captured at the temporal scale under BaP contamination. In addition, dispersion analysis was also performed, and the results indicated a higher level of dispersion among BaP-contaminated samples (BN and B microcosms). The results of Adonis analysis further confirmed that the effects of the treatment (BaP contamination and nitrate biostimulation) and incubation time on the microbial community structures were significant (*p* < 0.001) (Additional file 1: Table S1).

### Time-series network profiles of the mangrove microbial community under different treatments

**Fig. 4:**
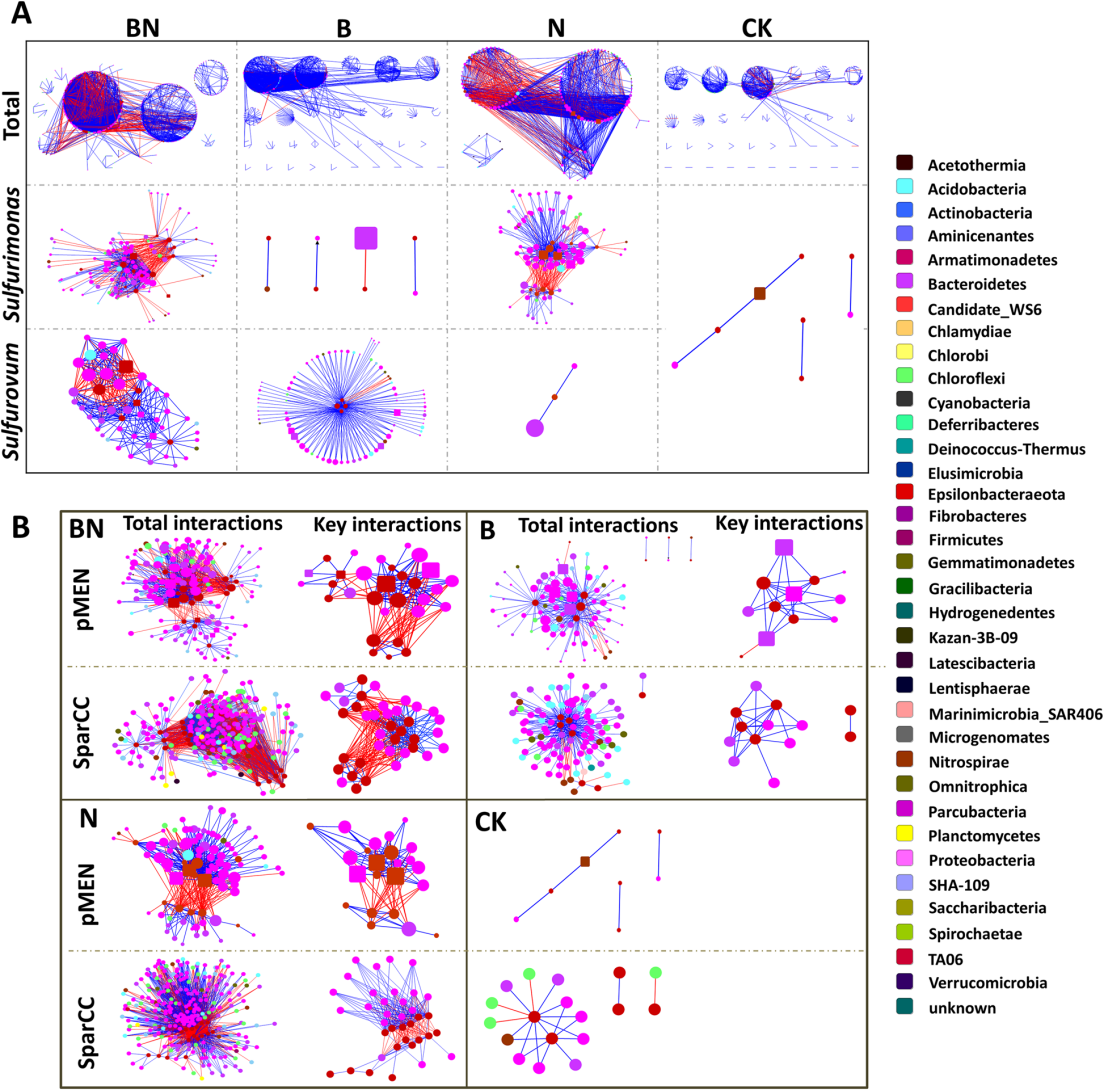
Time-series phylogenetic molecular ecological networks and interactions of specific genera (**A**) and their comparison to SparCC networks (**B**) under different treatments. Treatments include BaP and nitrate contamination (BN), BaP contamination (B), nitrate addition (N), and no-addition (CK). Each node signifies an OTU, and its color indicates the phylum to which it belongs. The keystone taxa were represented by rectangles. The sizes of the nodes are proportional to the node degree. A blue edge indicates a positive interaction between two individual nodes, while a red edge indicates a negative interaction

The OTUs from temporal scale samples (day 1, day 30, day 60, and day 90) under the same treatment were used to construct a time-series phylogenetic molecular ecological network. Four TMENs under different treatments were obtained (Fig. 4A), and their network indices are summarized in Additional file 1: Table S2.

B-TMEN had the largest network size, with 559 nodes and 1877 links. N-TMEN comprised the fewest members, with 174 nodes but many more links (1267 links) than CK-TMEN (742 links), while CK-TMEN had a larger network size (400 nodes). Comprised of moderate network size, BN-TMEN possessed maximum links. With respect to the indices relative to reachability and transfer efficiency of the network, N-TMEN possessed the largest value of average connectivity (avgK), average clustering coefficient (avgCC), and centralization of degree (CD), with the shortest harmonic geodesic distance (HD) and the smallest centralization of betweenness (CB) and stress centrality (CS), while CK-TMEN presented the opposite characteristics. In addition, BN-TMEN and B-TMEN ranked second and third in the values of avgK, avgCC, CD, CB, and CS and showed the reverse tendency in the HD value.

### Identification of keystone taxa and keystone guilds

**Fig. 5:**
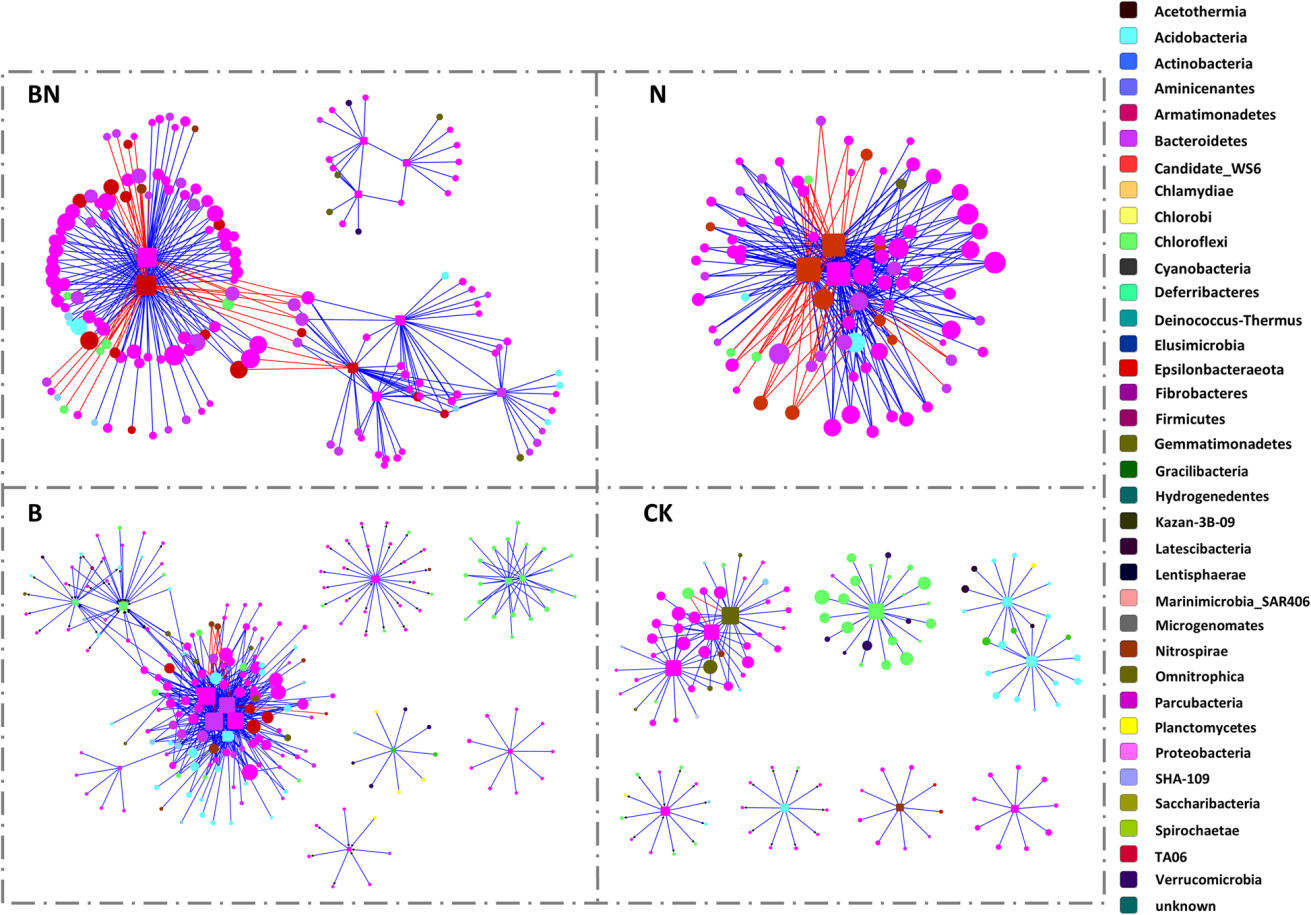
The keystone taxa identified and their links to neighbors in treated microcosms. Treatments include BaP and nitrate contamination (BN), BaP contamination (B), nitrate addition (N), and no-addition (CK)-treated microcosms. Each node signifies an OTU, and its color indicates the phylum to which it belongs. The keystone taxa were represented by rectangles. The sizes of the nodes are proportional to the node degree. A blue edge indicates a positive interaction between two individual nodes, while a red edge indicates a negative interaction

Based on within-module connectivity (Zi) and among-module connectivity (Pi), several keystone taxa were detected in each network (Fig. 5 and Additional file 1: Table S3). In total, 9, 3, 14, and 10 OTUs were identified as module hubs (Zi >  = 2.5) in BN-TMEN, N-TMEN, B-TMEN, and CK-TMEN, and 2 and 3 OTUs were identified as connectors (Pi ≥ 0.62) in N-TMEN and B-TMEN, respectively. Taxonomically, they belonged mainly to the phyla Proteobacteria (19), Bacteroidetes (5), Acidobacteria (5), and Chloroflexi (5). When mostly keystone taxa in CK-TMEN were affiliated with unknown bacteria, B-TMEN possessed the most diverse keystone taxa, such as *Novosphingobium*, *Robiginitalea*, *Prolixibacter*, and *Nitrospina Suflurovum*, was also a nearly keystone taxon with a larger Zi than those in CK-TMEN, approaching the threshold (Zi = 2.4). In contrast, keystone taxa in N-TMEN were affiliated mainly with *Sulfurimonas* and *Thioalkalispira*. In comparison, several keystone taxa that occurred in B-TMEN and N-TMEN maintained their keystone significance in BN-TMEN, including *Sulfurimonas*, *Thioalkalispra*, *Sulfurovum*, and *Robiginitalea*, indicating their consistent roles as keystone taxa under nitrate biostimulation and BaP contamination, respectively. *Sulfurimonas* and *Sulfurovum* occupy similar niches in the natural environment; here, their different responses and adaptations to BaP contamination and nitrate stimulation draw our attention.

The positions of *Sulfurimonas* and *Sulfurovum* in microbial networks and interactions with other members further revealed their role transitions under environmental factor changes and their extensive mutualism within keystone guilds (Figs. 4 and 5). In untreated microcosms, *Sulfurimonas* and *Sulfurovum* showed neutral interactions with each other or other members. Nitrate addition triggered the role transition of *Sulfurimonas*. They transited into keystone taxa in N-TMEN, when *Sulfurovum* was still barely present. Meanwhile, they interacted positively and significantly with another keystone taxon, *Thioalkalispira*, and formed mutualized guilds with other microbes, such as *Sedimenticola* and *Thiobacillus*. However, under BaP contamination, *Sulfurovum* showed significance, and their mutualization within a keystone guild was frequently detected. The keystone guild was dominated by multiple keystone taxa, including *Novosphingobium*, *Robiginitalea*, and *Prolixibacter*. In BN-TMEN, *Sulfurimonas* and *Sulfurovum* were found to simultaneously act as keystone taxa and formed keystone guilds in similar synergistic patterns as in N-TMEN and B-TMEN, respectively. In addition, these two guilds competed with each other, mainly via *Sulfurimonas* and *Thioalkalispira* against *Sulfurovum*.

To verify the robustness of the detected connections and keystone taxa, a permutation-based SparCC approach was also performed to construct networks and identify keystone taxa (Additional file 1: Tables S2 and S4). The results showed that the numbers of nodes and links of TMENs were different from those of SparCC networks (SNs), which may be caused by the different selection strategies of correlation values. However, the keystone significance of *Sulfurimonas* and *Sulfurovum* and their role transitions in SNs were similar to those of TMENs across different treatments, not only in their interactions with each other but also in their mutualism with other key members, such as *Thioalkalispira*, *Novosphingobium*, and *Robiginitalea* (Fig. 4B).

### Comparative genomics of Sulfurovum and Sulfurimonas

To obtain further insight into the genomic traits of *Sulfurovum* from *Sulfurimonas* populations, we selected and compared the genomic features of 12 *Sulfurovum* and 14 *Sulfurimonas* whole genomes or metagenomic fragment assembled genomes (*Sulfurovum* AG and *Sulfurimonas* AG in short) from public databases. An overview of these genomes is shown in Additional file 1: Table S5.

**Fig. 6:**
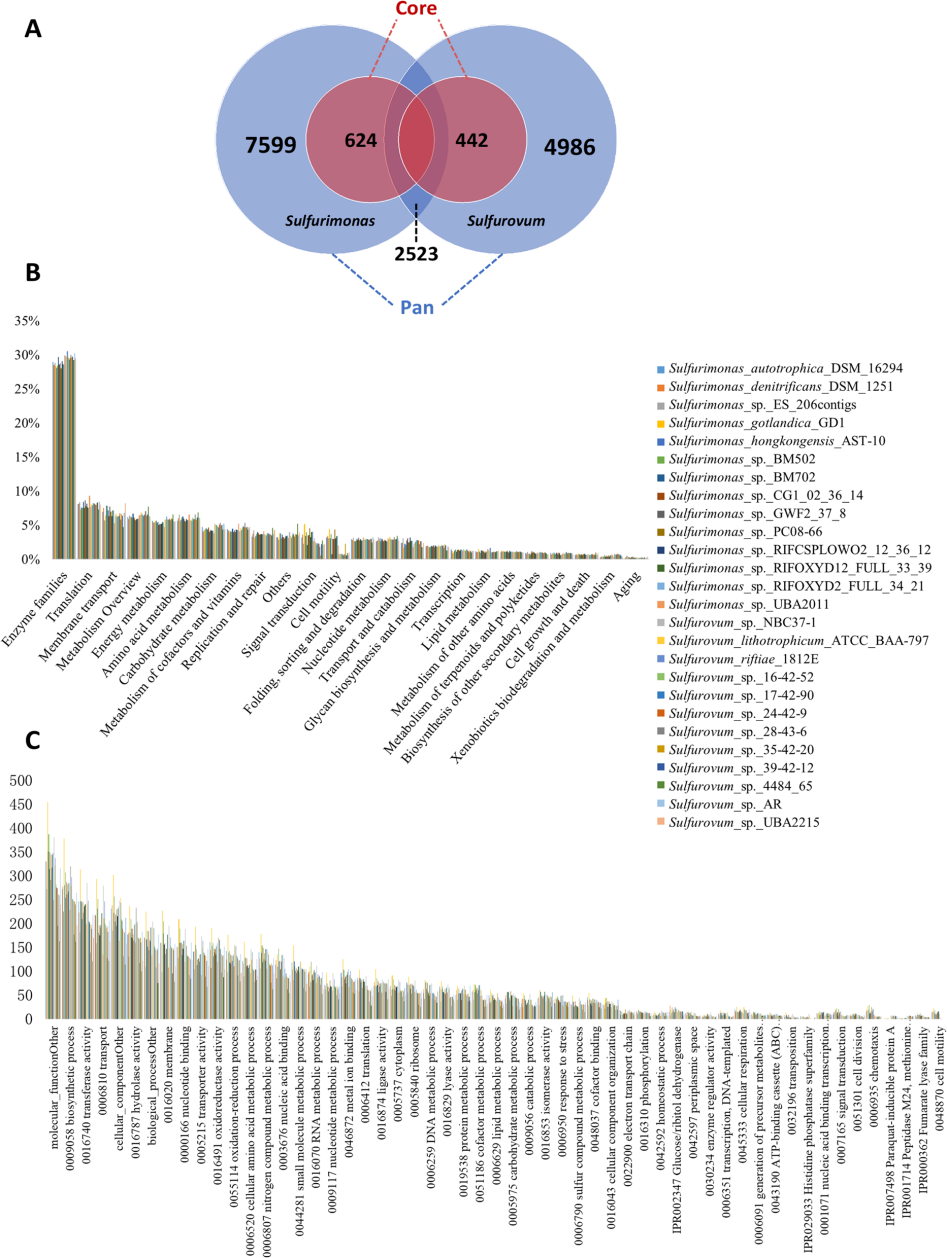
Pangenomic analysis of *Sulfurimonas* AGs and 12 *Sulfurovum* AGs. **A** The size of homolog families clustered by genes in the pangenomes and core genomes of *Sulfurimonas* AGs and *Sulfurovum* AGs and their shared homolog families. **B** The functional categories annotated against the KEGG database. **C** The top 55 functional categories annotated against the Gene Ontology (GO) database

To elucidate genus-level potential metabolic traits, pangenome calculations were conducted on the Kbase platform. Regarding the pangenome of *Sulfurovum* AG (*Sulfurovum* Pan), 24,022 genes were clustered in total, where 21,792 genes were clustered into 2935 homolog families and 2230 were in singleton families. For the pangenome of *Sulfurimonas* AG (*Suflurimonas* Pan), 32,095 genes were detected in total, where 27,937 genes were clustered into 3703 homolog families and 4158 were in singleton families. As shown in Table S5, the total number of genes per *Sulfurovum* genome was 2002 on average, which was significantly less than the total number of genes per *Sulfurimonas* (*p* < 0.05), but their number of homologs was comparable, and singleton genes were significantly different (*p* < 0.05). Comparing *Sulfurovum* Pan and *Sulfurimonas* Pan (Fig. 6A), 50.6% and 33.2% of homologous families were shared between them. These two pangenomes have core gene sets of 442 and 642 with comparable sizes.

Functional annotation of the genomes was performed against the specialized KEGG and GO term databases (Fig. 6B, C). The functional categories of enzyme families predominated, followed by translation, membrane transport, energy metabolism, amino acid metabolism, and carbohydrate metabolism. With respect to GO annotation, the genes belonging to biosynthesis process, transferase activity, transport, and hydrolase activity ranked highest. At higher resolution, more than one fermentation pathway was discovered in *Sulfurovum* Pan, including those generating hydrogen, acetate, and succinate. In addition, LysE homology involving the export of arginine and lysine and omcB homology participating in extracellular electron transfer were also detected in *Sulfurovum* Pan.

**Fig. 7:**
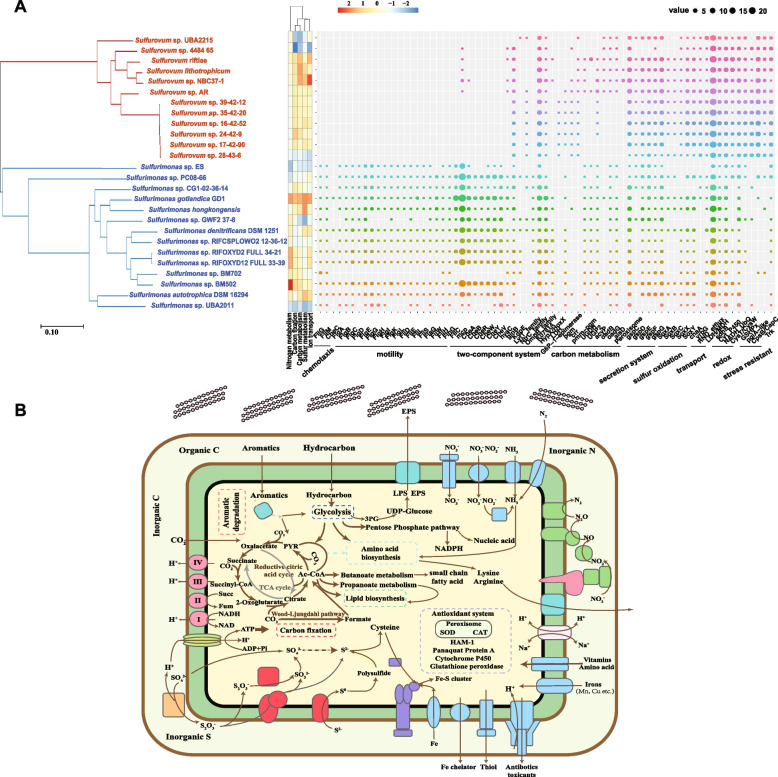
**A** Phylogenetic tree of *Sulfurimonas* and *Sulfurovum* representatives and their significantly overrepresented functions; **B** predicted metabolic pathway scheme based on *Sulfurovum* Pan and MAGs. The phylogenetic tree in the left panel was based on a set of 49 core universal genes defined by COG (clusters of orthologous groups) gene families in 14 *Sulfurimonas* and 12 *Sulfurovum* representative genomes. The significantly overrepresented KEGG functional categories at level 3 and GO functional categories at level 2 are shown in the middle, and the genes in key biological processes whose abundances were significantly different in the two genera are shown in the right panel

To identify possible functional divergences of *Sulfurovum* AG from *Sulfurimonas* AG, further significant difference analysis was performed (Fig. 7A). All the annotated results against different databases indicated that cell motility and signal transduction were sharply overrepresented in *Sulfurimonas* genomes, while other significantly different categories were mainly overrepresented in *Sulfurovum* genomes, such as carbohydrate metabolism, amino acid metabolism, cofactor and vitamin metabolism, and transport activity. For the higher resolution of functional categories, KEGG results indicated the enrichment of genes related to bacterial motility protein, chemotaxis, two-component system, and flagellar assembly in *Sulfurimonas*. Genes related to enzymes; carbon metabolic pathways, such as glycolysis, carbon fixation, and the pentose phosphate pathway; secretion system; and peroxisome were overrepresented in *Sulfurovum*. Regarding GO terms, similar overrepresented categories were discovered in *Sulfurimonas*, along with the enrichment of nitrogen metabolism, while transporter activity, oxidation–reduction process, hydrolase and ligase activity, paraquat-inducible protein A, lipid polysaccharide export, and sulfur metabolism were overrepresented in *Sulfurovum*.

### Genomic traits of Sulfurovum and Sulfurimonas genomes recruited from microcosm metagenomes

Based on metagenomes from microcosms under BN, B, N, and CK treatments, we binned and refined 4 MAGs affiliated with *Sulfurovum* and 2 MAGs affiliated with *Sulfurimonas* (Additional file 1: Table S6). We obtained only 2 qualified *Sulfurimonas* MAGs from the BN and N metagenome datasets, and their completeness was 81.33 and 51.16, with contamination rates of 4.29 and 1.72%, respectively. These *Sulfurimonas* MAGs exhibited similar metabolic potential to other *Sulfurimonas* representative genomes based on KEGG pathways and clusters of orthologous genes (COG) annotations. For *Sulfurovum* MAGs, their completeness ranged from 70.33 to 81.52, with a 7.18–14.66 contamination rate. Compared to other representative *Sulfurovum* genomes, these *Sulfurovum* MAGs possess similar metabolic potential, except for a significantly higher abundance of the COG categories RNA processing and modification and inorganic ion transport and metabolism, and the genes coding nitrogenase were also found in mangrove MAGs, which are absent in *Sulfurovum* derived from other habitats.

### Co-culture of isolated BaP-degrading strain with Sulfurovum and Sulfurimonas type strains

To verify whether *Sulfurovum/Sulfurimonas* assist its connected keystone taxa and explore its role in the mangrove microbiome responding to BaP contamination, co-culture experiments were launched. Since OTU5484 was the only keystone taxon affiliated with the known PAH degrader *Novosphingobium* and involved in keystone guilds, it may be the key degrader in the mangrove microbiome. hiTAIL PCR was performed to define taxonomic information at the species/strain level by obtaining nearly complete 16S rRNA gene sequences. The results showed that 16S rRNA gene sequences with lengths ranging from 851–1228 bp were generated, and most of them had the closest phylogenetic relationships with *N. pentaromativorans* US6-1 (the identity is 95.57–100%), which is capable of BaP degradation (Additional file 1: Table S7). Thus, *N. pentaromativorans* US6-1 was used as the key player for BaP degradation to construct co-culture systems with *Sulfurovum/Sulfurimonas* strains*.*

Among the few pure cultures of *Sulfurovum* and *Sulfurimonas*, the type strains of *Sulfurovum indicum* and *Sulfurimonas indica* were isolated from the same habitat occupying the same niche and are therefore suitable for comparison. However, their genomic contents were comparable to those of their interspecies: when *Sulfurovum indicum* possesses abundant genes involved in transport and antioxidant enzymes, *Sulfurimonas indica* is enriched in genes related to chemotaxis and motility (Additional file 1: Fig. S2). Thus, these two strains were selected for the co-culture experiment with US6-1 under BaP contamination. To examine whether *Sulfurovum indicum* and *Sulfurimonas indica* tolerate BaP, their growth with/without BaP addition was measured, and both grew well under BaP contamination and reached the highest biomass at 48 and 24 h, respectively (Additional file 1: Fig. S2).

**Fig. 8:**
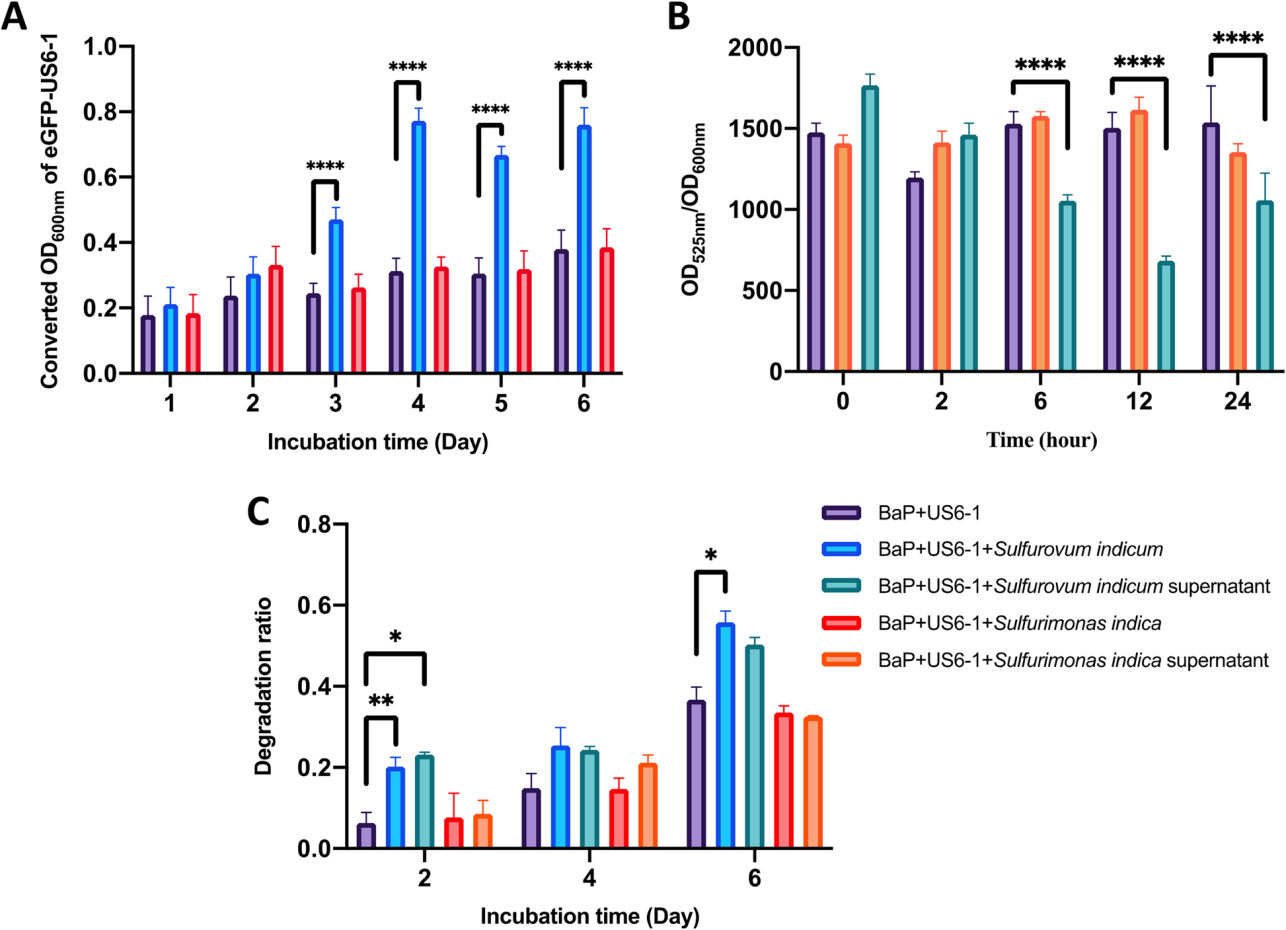
The biomass of eGFP-US6-1 calculated according to the fluorescence intensity (**A**), ROS level (**B**), and BaP degradation rate (**C**) of co-culture and mono-culture systems. Asterisks indicate statistically significant (*p < 0.05; **p < 0.01; ****p < 0.0001) differences based on two-way ANOVA with the Geisser-Greenhouse correction

Then, co-culture systems were constructed, and the growth of eGFP-US6-1 was determined according to the fluorescence intensity (Fig. 8A; the standard curve between fluorescence intensity and OD_600nm_ is shown in Additional file 1: Fig. S3). The results showed that *Sulfurovum indicum* significantly promoted the growth of eGFP-US6-1 under BaP contamination after 3 days compared to controls and co-cultures with *Sulfurimonas indica* (*p* < 0.0001) and reached the highest biomass on day 4 (OD_600nm_ = 0.77). The measurement of ROS levels in supernatant-based co-cultures further indicated that extracellular products of *Sulfurovum indicum* decreased the ROS level of US6-1 significantly from 6 h–24 h (*p* < 0.0001) (Fig. 8B). Both cell- and supernatant-based co-culture with *Sulfurovum indicum* enhanced the BaP degradation rate, from 6.3 to 20.2% (*p* < 0.01) and 23.1% (*p* < 0.05) on day 2 and from 36.7 to 55.8% (*p* < 0.05) and 50.3% on day 6 (Fig. 8C).

## Discussion

Identifying keystone taxa and revealing their ecological, structural, and functional roles in driving microbiome structure and function is a critical but difficult issue. The present study aimed to find effective solutions to this issue, taking keystone taxa in BaP-degrading keystone guilds as a case study.

Facing the sudden input of BaP and/or nitrate, mangrove microorganisms significantly altered their communities: when nitrate replenishment decreased the microbial diversity to a large extent, BaP tended to stimulate more complex and multidirectional alteration of the microbial community structures according to the higher dispersion degree among BaP-contaminated samples. This finding was consistent with the results of network analysis, which showed that the fewest bacteria were involved in N-TMEN but with the highest efficiency of information transfer (largest avgK, avgCC, and shortest HD) [37], and B-TMEN possessed the largest network size. At the same time, obvious turnover of keystone taxa associated with different treatments was observed. These results inferred that changes in environmental factors strongly drive the reassembly of mangrove microbial community structure and trigger the role transition of functional keystone taxa.

In microbial association networks, multiple keystone taxa in different treatment microcosms were identified; therein, *Sulfurovum* was selected as the prime example with similar niche-occupied *Sulfurimonas* as a reference, since they showed well-defined and differentiated role transitions. *Sulfurovum* and *Sulfurimonas* are famous sulfur-oxidizing and nitrate-reducing bacteria (SONRB). They come from the phylum Epsilonbacteraeota, and both cultivation-dependent and cultivation-independent studies detected their dominance and co-occurrence across habitats where strong gradients of oxygen and sulfide exist [38–40]. Some genomics studies described their similar metabolic versatile utilization of sulfur, nitrogen compounds [41], and oxygen preference [40]. Thus, although these two SONRBs are phylogenetically distinct, *Sulfurovum* and *Sulfurimonas* seem to share similar niches in natural environments. However, the present study demonstrated their distinct role transitions: *Sulfurimonas* was dominant in sulfide-rich mangrove sediments and transited into keystone taxa under nitrate addition samples (BN-TMEN and N-TMEN), while *Sulfurovum* was absent from N-TMEN but active in BaP-contaminated samples (BN-TMEN and B-TMEN) and exhibited significant competition with *Sulfurimonas* in BN-TMEN.

Notably, *Sulfurimonas* and *Sulfurovum* interacted with other keystone taxa and formed keystone guilds under nitrate addition and BaP contamination. Keystone guilds consist of multiple interacting keystone taxa and may result from resource sharing, nutrient complementary acquisition, niche partitioning, and spatiotemporal coherence and have a greater influence on a broad process [9]. When *Sulfurimonas* mainly formed a keystone guild with *Thioalkalispira* in nitrate addition microcosms, *Sulfurovum* mutualized in densely PAH-degrader-centric keystone guilds with the PAH degrader *Novosphingobium* [42] in BaP-contaminated samples (B-TMEN and BN-TMEN), where 58.90% and 63.25% of added BaP were removed. Their formation and different assembly under environmental changes indicated the significance and possible mechanism of how keystone taxa orchestrate microbiome structure and function.

A comparative genomics approach was further employed to provide the genetic basis for the role transitions of *Sulfurovum* and *Sulfurimonas* to characterize these two keystone taxa in their respective keystone guilds. Much fewer but sharper functional categories were overrepresented in *Sulfurimonas* AG, including mainly bacterial motility (flagellar assembly) and chemotaxis (two-component system), suggesting that its quicker response and motility to stimuli (nitrate) could be the reason for them standing out from competition under nitrate replenishment conditions with highly efficient use of nitrate [43, 44].

Compared to *Sulfurimonas*, more diverse functions were overrepresented in *Sulfurovum* AG. With respect to carbon metabolism and energy generation, the genes related to glycolysis, the central tricarboxylic acid (TCA) cycle, pentose phosphate pathway, downstream butanoate, and propanoate metabolism and Sox pathway were all more frequent in *Sulfurovum* genomes. This process could be an advantage for their survival in organic contaminated environments, considering that these pathways facilitated the replenishment of polysaccharides, nucleic acids, and short-chain fatty acids [45] and may generate more ATP and NADPH to support carbon fixation [46]. Thus, thriving carbon metabolism and energy generation may be a genetic basis for *Sulfurovum* to outcompete *Sulfurimonas* under BaP contamination, even taking a role in the BaP polymicrobial degradation network.

Better resistance to oxidative stress is another potential advantage for *Suflurovum* under BaP contamination. PAHs are usually oxidized to oxygenated intermediates and cause the overproduction of ROS, resulting in oxidative stress [47]. Organisms always regulate ROS levels by a number of enzymatic and buffering processes, generally involving superoxide dismutase (SOD), catalase (CAT), glutathione peroxidase, and cytochrome P450 [48], which are overrepresented in *Sulfurovum*. In addition, the gene encoding paraquat-inducible protein A was also overrepresented, which could be inducible by superoxide generators [49]. In addition to regulating the system against oxidative stress themselves, pangenomics analysis of *Suflurovum* (Fig. 7B) further detected the presence of the genes involved in biofilm formation, generation of iron chelators and thiols, the exporter of polysaccharides, and resistance-nodulation-division (RND) efflux pumps, which potentially assist other microbes in buffering oxidative stress by scavenging ROS, exchanging information, providing protection for biofilm formation and releasing cell-penetrating compounds, etc. [50].

In the gene pools of *Sulfurovum*, transport activity (transporter) was significantly overrepresented, and large amounts of transporters involved in the transport of vitamins, amino acids, and inorganics were revealed. Therein, the lysine-type exporter protein LysE was identified, which is involved in the export of arginine and lysine. These biosynthetically costly amino acids seem to promote stronger cooperative interactions [8] by providing precious nutrients to other microbes. In addition, the presence of omcB homology in *Sulfurovum* Pan indicated the potential to pump out electrons for redox processes and promote BaP degradation, as omcB has a major role in electron transfer to extracellular Fe via the outer membrane [51], in accordance with previous studies demonstrating the ability of SOB to shuttle electrons from reduced sulfur compounds to extracellular electron acceptors [52, 53]. Most *Sulfurovum* genomes also possess the acetic acid fermentation pathway, which allows them to generate acetic compounds, which are reported to have potential roles in the resuscitation of microbes from the viable but nonculturable (VBNC) state [54].

As stated, comparative genomics suggested the genetic basis of *Sulfurovum* to exert influence on microbiome functioning with advantages in carbon catabolism, biofilm formation, oxidation resistance, and nutrient cross-feeding. Considering that genes only work under given conditions, and comparative network analysis illustrated that *Sulfurovum* likely functioned in BaP-degrading keystone guilds, in vitro co-culture experiments were launched to further verify their functional role in simulating the microbe-to-microbe interactions in this guild. With the help of the hiTAIL-PCR technique, the key BaP degrader US6-1 in this functional guild was captured and regarded as the key player in BaP degradation, while the absence of BaP degradation pathways in *Sulfuorovum* genomes indicated their helper role in the BaP degradation process. In addition, as occupying niches similar to those of *Sulfurovum* in natural environments, *Sulfurimonas* exhibited entirely different responses to BaP and nitrate input and was absent in BaP-degrading keystone guilds, making it a suitable reference in co-culture experiments. Then, the results not only proved the tolerance of *Sulfurovum* under BaP stress but also confirmed that they help US6-1 reduce ROS levels by extracellular products and promote US6-1 growth prominently, as well as improve the BaP degradation efficiency, experimentally demonstrating that *Sulfurovum* could facilitate the other members against BaP toxicity by scavenging ROS and providing nutrients, ultimately promoting the BaP degradation process.

## Conclusions

Our study drew a clear trajectory for the role transitions of two similar-niche-occupying SONRB *Sulfurimonas* and *Sulfurovum* and their participation in the assembly of synergistic keystone guilds under nitrate stimulation or BaP contamination, indicative of the powerfulness of co-occurrence network analysis in inferring keystone taxa/guild. Comparative genomics can further provide genetic evidence for the functional roles of keystone taxa when co-culture of targeted capturing keystone taxa experimentally verified the working activity of *Sulfurovum* in the BaP degrading keystone guild. With the proposed “3C-strategy,” our findings highlight the significance of keystone guilds as superior functional units, and it is important to place keystone taxa into the functional guild they live in, which makes them target-characterized and experimentally tractable when exploring their ecological and functional roles. Meanwhile, the key players in BaP degradation and the interaction pattern of the BaP-degrading keystone guild captured in the present study provided important clues to construct functional communities for pollutant removal. Larger longitudinal studies are needed to evaluate the generality of basic ecological concepts of microbial keystone taxa as applied to the environmental microbiome, but the present study provides an alternative solution for the characterization and manipulation of keystone taxa in cases facing sudden environmental factor changes, such as mangrove sediment microbiomes suffering from anthropogenic contaminant inputs, marine microbiomes at oil-spill sites, plant microbiomes being exposed to overused agricultural chemicals or pathogens, and even human gut microbiomes disrupted by antibiotics, disease, or diet-mediated interventions.

## Supplementary Information

**Additional file 1: Fig. S1.** The phylogenetic α-diversity index values of microbial communities in microcosms including (A) Faith’s PD, (B) Rao’s Entropy and (C) mean phylogenetic distance (MPD). **Fig. S2.** The growth of *Sulfurovum indicum *(A) and *Sulfurimonas indica* (B) with or without BaP addition, and the main shared/unique genes in their genomes (C). **Fig. S3.** The regression equation for fluorescence intensity and OD_600nm_ of eGFP-US6-1. **Table S1.** Significance of the effects of different treatments on the microbial community structure using Adonis analysis. **Table S2.** Topological properties of the time-series phylogenetic molecular ecological networks (TMENs) and SparCC networks (SN). **Table S3.** Keystone taxa of the time-series phylogenetic molecular ecological networks under different treatments. The keystone taxa also identified in the SparCC networks were marked in red. **Table S4.** Keystone taxa of the SparCC networks under different treatments. The keystone taxa also identified in the time-series phylogenetic molecular ecological networks were marked in red. **Table S5.** General genomic features of genomes in this study relative to *Sulfurimonas* and *Sulfurovum*. **Table S6.** The completeness and contamination of *Sulfurimonas* and *Sulfurovum* MAGs. **Table S7.** The 16S rRNA gene sequences generated by hiTAIL PCR and their closest phylogenetic affiliation obtained using Blast.

## Data Availability

The raw sequence data (including 16S rRNA gene amplicons and shotgun metagenomes under Experiment ID OEX012584 and OEX012587) and assemblies generated from metagenomes (analysis ID OEZ014090-OEZ014094) in the current study are available in the NODE repository (https://www.biosino.org/node/index) under project ID OEP001343. *Sulfurovum* and *Sulfurimonas* MAGs have been deposited in eLMSG (https://www.biosino.org/elmsg/index) under accession numbers LMSG_G000026856.1-LMSG_G000026861.1.

## Abbreviations

BaP: Benzo[a]-pyrene
PAH: Polycyclic aromatic hydrocarbon
MAGs: Metagenomic assembled genomes
hiTAIL-PCR: High-efficiency thermal asymmetric interlaced PCR
eGFP: Enhanced green fluorescent protein
ROS: Reactive oxygen species
HMW-PAHs: High molecular weight polycyclic aromatic hydrocarbons
SONRB: Sulfur oxidizing and nitrate reducing bacteria
pMENs: Phylogenetic molecular ecological networks
TMEN: Time-series pMENs
SparCC: Sparse Correlations for Compositional data
MPD: Mean phylogenetic distance
avgK: Average connectivity
avgCC: Average clustering coefficient
CD: Centralization of degree
HD: Harmonic geodesic distance
CB: Centralization of betweenness
CS: Stress centrality
COGs: Clusters of orthologous groups
*Sulfurimonas* AG: *Sulfurimonas* Whole genomes or metagenomic fragment assembled genomes
*Sulfurovum* AG: *Sulfurovum* Whole genomes or metagenomic fragment assembled genomes
*Suflurimonas* Pan: Pan-genome of *Sulfurimonas* AG
*Sulfurovum* Pan: Pan-genome of *Sulfurovum* AG
SOD: Superoxide dismutase
CAT: Catalase
EPS: Extracellular polymeric substances
RND efflux pumps: Resistance-nodulation-division efflux pumps
VBNC: Viable but non-culturable
