# Supplementary material for: Characterization of two keystone taxa, sulfur-oxidizing, and nitrate-reducing bacteria, by tracking their role transitions in the benzo[a]pyrene degradative microbiome
Source: Microbiome. 2023 Jun 24;11:139. doi: 10.1186/s40168-023-01583-1 (PMC10290299; doi:10.1186/s40168-023-01583-1)
Supplement: Supplementary file 2 — Additional file 1: Fig. S1. The phylogenetic α-diversity index values of microbial communities in microcosms including (A) Faith’s PD, (B) Rao’s Entropy and (C) mean phylogenetic distance (MPD). Fig. S2. The growth of Sulfurovum indicum (A) and Sulfurimonas indica (B) with or without BaP addition, and the main shared/unique genes in their genomes (C). Fig. S3. The regression equation for fluorescence intensity and OD600nm of eGFP-US6-1. Table S1. Significance of the effects of different treatments on the microbial community structure using Adonis analysis. Table S2. Topological properties of the time-series phylogenetic molecular ecological networks (TMENs) and SparCC networks (SN). Table S3. Keystone taxa of the time-series phylogenetic molecular ecological networks under different treatments. The keystone taxa also identified in the SparCC networks were marked in red. Table S4. Keystone taxa of the SparCC networks under different treatments. The keystone taxa also identified in the time-series phylogenetic molecular ecological networks were marked in red. Table S5. General genomic features of genomes in this study relative to Sulfurimonas and Sulfurovum. Table S6. The completeness and contamination of Sulfurimonas and Sulfurovum MAGs. Table S7. The 16S rRNA gene sequences generated by hiTAIL PCR and their closest phylogenetic affiliation obtained using Blast. [file 40168_2023_1583_MOESM1_ESM.docx]

**Characterization of two keystone taxa, sulfur-oxidizing and nitrate-reducing bacteria, by tracking their role transitions in the benzo[a]pyrene degradative microbiome**

Xiaolan Lin^1^, Baoyi Qiao^1^, Ruirui Chang^1^, Yixin Li^1^, Wei Zheng^1^, Zhili He^3^, Yun Tian^1,2**^

**^1^**Key Laboratory of the Ministry of Education for Coastal and Wetland Ecosystems, School of Life Sciences, Xiamen University, Xiamen 361102, China

**^2^**State Key Laboratory of Marine Environmental Science, Xiamen University, Xiamen 361102, China

**^3^** Southern Marine Science and Engineering Guangdong Laboratory (Zhuhai), Zhuhai 519080, China

**Corresponding author: [tianyun@xmu.edu.cn](mailto:tianyun@xmu.edu.cn) (Y Tian)

**Supplementary information**

**Supplementary figures**

**Fig. S1 The phylogenetic α-diversity index values of microbial communities in microcosms including (A) Faith’s PD, (B) Rao’s Entropy and (C) mean phylogenetic distance (MPD)**

**Fig. S2 The growth of *Sulfurovum indicum* (A) and *Sulfurimonas indica* (B) with or without BaP addition, and the main shared/unique genes in their genomes (C).**

**Fig. S3 The regression equation for fluorescence intensity and OD_600nm_ of eGFP-US6-1**

**Supplementary tables**

**Table S1 Significance of the effects of different treatments on the microbial community structure using Adonis analysis**

**Table S2 Topological properties of the time-series phylogenetic molecular ecological networks (TMENs) and SparCC networks (SN)**

**Table S3 Keystone taxa of the time-series phylogenetic molecular ecological networks under different treatments. The keystone taxa also identified in the SparCC networks were marked in red.**

**Table S4 Keystone taxa of the SparCC networks under different treatments. The keystone taxa also identified in the time-series phylogenetic molecular ecological networks were marked in red.**

**Table S5 General genomic features of genomes in this study relative to *Sulfurimonas* and *Sulfurovum***

**Table S6 The completeness and contamination of *Sulfurimonas* and *Sulfurovum* MAGs**

**Table S7** **The 16S rRNA gene sequences generated by hiTAIL PCR and their closest phylogenetic affiliation obtained using Blast**

**Fig. S1 The phylogenetic α-diversity index values of microbial communities in microcosms including (A) Faith’s PD, (B) Rao’s Entropy and (C) mean phylogenetic distance (MPD)**

**
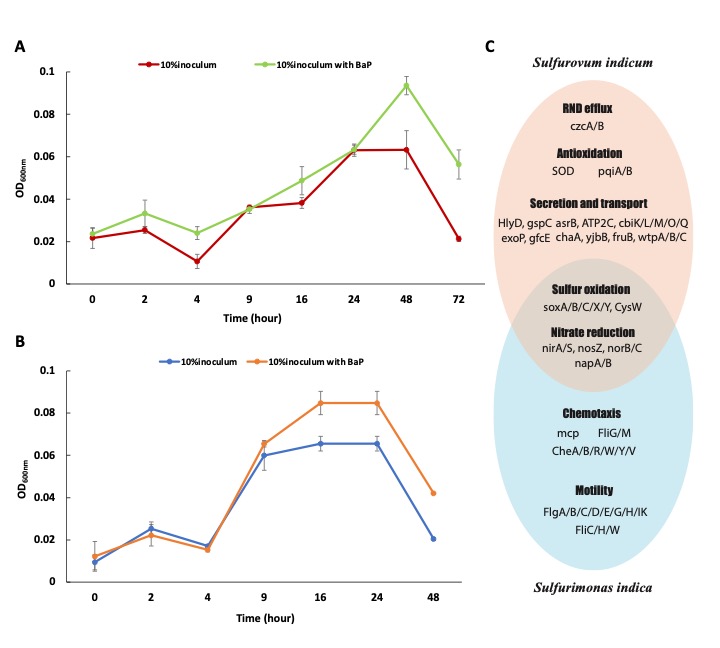
**

**Fig. S2 The growth of *Sulfurovum indicum* (A) and *Sulfurimonas indica* (B) with or without BaP addition, and the main shared/unique genes in their genomes (C).**

**Fig. S3 The regression equation for fluorescence intensity and OD_600nm_ of eGFP-US6-1**

**Table S1** **Significance of the effects of different treatments on the microbial community structure using Adonis analysis**

| **Group** | ***F*** | **R^2^** | ***P*** | **significance** |
| --- | --- | --- | --- | --- |
| **treatment** | 48.389 | 0.49451 | 0.001 | *** |
| **day** | 5.716 | 0.05842 | 0.001 | *** |
| **treatment: day** | 2.374 | 0.04852 | 0.002 | ** |
|  |  |  |  |  |
| **BaP** | 2.1602 | 0.03591 | 0.002 | ** |

**Table S2 Topological properties of the time-series phylogenetic molecular ecological networks (TMENs) and SparCC networks (SN)**

| **Network Indexes** | **BN-TMEN** | **N-TMEN** | **B-TMEN** | **CK-TMEN** |
| --- | --- | --- | --- | --- |
| Total nodes | 413 | 174 | 559 | 400 |
| Total links | 2232 | 1267 | 1877 | 742 |
| R square of power-law | 0.797 | 0.642 | 0.827 | 0.937 |
| Average degree (avgK) | 10.809 | 14.563 | 6.716 | 3.71 |
| Average clustering coefficient (avgCC) | 0.324 | 0.439 | 0.222 | 0.206 |
| Average path distance (GD) | 3.674 | 2.354 | 3.811 | 5.895 |
| Geodesic efficiency (E) | 0.339 | 0.501 | 0.321 | 0.221 |
| Harmonic geodesic distance (HD) | 2.948 | 1.995 | 3.115 | 4.533 |
| Maximal degree | 92 | 76 | 83 | 27 |
| Centralization of degree (CD) | 0.198 | 0.359 | 0.137 | 0.059 |
| Maximal betweenness | 6404.399 | 761.896 | 7861.653 | 12416.297 |
| Centralization of betweenness (CB) | 0.073 | 0.047 | 0.049 | 0.151 |
| Maximal stress centrality | 114170 | 5871 | 136714 | 118666 |
| Centralization of stress centrality (CS) | 1.293 | 0.353 | 0.861 | 1.442 |
| Density (D) | 0.026 | 0.084 | 0.012 | 0.009 |
| Transitivity (Trans) | 0.518 | 0.511 | 0.442 | 0.374 |
| Connectedness (Con) | 0.486 | 0.555 | 0.338 | 0.459 |
| **Network Indexes** | **BN-SN** | **N-SN** | **B-SN** | **CK-SN** |
| Total nodes | 526 | 450 | 283 | 276 |
| Total links | 11184 | 11339 | 2048 | 951 |

**Table S3 Keystone taxa of the time-series phylogenetic molecular ecological networks under different treatments. The keystone taxa also identified in the SparCC networks were marked in red.**

| **Roles** | **ID** | **Zi** | **Pi** | **Phylum** | **Class** | | **Order** | **Family** | | **Genus** | | | |
| --- | --- | --- | --- | --- | --- | --- | --- | --- | --- | --- | --- | --- | --- |
| BN | | | | | | | | | | | | |  |
| module hub | OTU778 | 2.568912 | 0 | Bacteroidetes | Bacteroidia | | Flavobacteriales | Flavobacteriaceae | | *Robiginitalea* | | | |
|  | OTU827 | 3.785765 | 0.0768 | Proteobacteria | Gammaproteobacteria | | Cellvibrionales | Halieaceae | | OM60(NOR5) clade | | | |
|  | OTU4893 | 3.017828 | 0 | Proteobacteria | Deltaproteobacteria | | Deltaproteobacteria Incertae Sedis | Unknown | | *Deferrisoma* | | | |
|  | OTU5228 | 2.542579 | 0 | Proteobacteria | Deltaproteobacteria | | NB1-j | uncultured | |  | | | |
|  | OTU5792 | 2.501433 | 0.185679 | Epsilonbacteraeota | Campylobacteria | | Campylobacterales | Thiovulaceae | | *Sulfurimonas* | | | |
|  | OTU7481 | 2.771721 | 0.095 | Proteobacteria | Gammaproteobacteria | | B2M28 | uncultured | |  | | | |
|  | OTU9289 | 2.97453 | 0.35503 | Epsilonbacteraeota | Campylobacteria | | Campylobacterales | Sulfurovaceae | | *Sulfurovum* | | | |
|  | OTU11125 | 2.542579 | 0 | Proteobacteria | Deltaproteobacteria | | Desulfobacterales | Desulfobacteraceae | | SEEP-SRB1 | | | |
|  | OTU13057 | 2.501433 | 0.091073 | Proteobacteria | Gammaproteobacteria | | Ectothiorhodospirales | Thioalkalispiraceae | | *Thioalkalispira* | | | |
| N | | | | | | | | | | | | |  |
| module hub | OTU5792 | 2.617192 | 0.546745 | Epsilonbacteraeota | Campylobacteria | Campylobacterales | | | Thiovulaceae | | *Sulfurimonas* |  |  |
|  | OTU12381 | 2.534205 | 0.525714 | Epsilonbacteraeota | Campylobacteria | Campylobacterales | | | Thiovulaceae | | *Sulfurimonas* |  |  |
|  | OTU13057 | 2.617192 | 0.532933 | Proteobacteria | Gammaproteobacteria | Ectothiorhodospirales | | | Thioalkalispiraceae | | *Thioalkalispira* |  |  |
| connector | OTU6227 | -0.341 | 0.623457 | Proteobacteria | Gammaproteobacteria | Ectothiorhodospirales | | | Thioalkalispiraceae | | |  |  |
|  | OTU10110 | 1.704986 | 0.6216 | Bacteroidetes | Bacteroidia | Bacteroidales | | | Bacteroidetes BD2-2 | | uncultured |  |  |
| B | | | | | | | | | | | | |  |
| module hub | OTU8880 | 5.435909 | 0.384444 | Chloroflexi | Anaerolineae | | SBR1031 | uncultured | | | | | |
|  | OTU1462 | 5.089743 | 0 | Proteobacteria | Gammaproteobacteria | | Ectothiorhodospirales | Ectothiorhodospiraceae | | *Thiogranum* | | | |
|  | OTU778 | 3.897394 | 0.487734 | Bacteroidetes | Bacteroidia | | Flavobacteriales | Flavobacteriaceae | | *Robiginitalea* | | | |
|  | OTU13779 | 3.475866 | 0 | Proteobacteria | Gammaproteobacteria | | Tenderiales | Tenderiaceae | | *Candidatus* Tenderia | | | |
|  | OTU10062 | 3.32339 | 0 | Chloroflexi | Anaerolineae | | Anaerolineales | Anaerolineaceae | | uncultured | | | |
|  | OTU5873 | 3.313477 | 0.214533 | Chloroflexi | Anaerolineae | | SBR1031 | uncultured | |  | | | |
|  | OTU5484 | 3.097353 | 0.5216 | Proteobacteria | Alphaproteobacteria | | Sphingomonadales | Sphingomonadaceae | | *Novosphingobium* | | | |
|  | OTU1644 | 2.862343 | 0 | Nitrospinae | Nitrospinia | | Nitrospinales | Nitrospinaceae | | *Nitrospina* | | | |
|  | OTU3093 | 2.85846 | 0 | Chloroflexi | Anaerolineae | | Anaerolineales | Anaerolineaceae | | uncultured | | | |
|  | OTU9530 | 2.823063 | 0.505113 | Bacteroidetes | Bacteroidia | | Bacteroidales | Prolixibacteraceae | | *Prolixibacter* | | | |
|  | OTU3001 | 2.797338 | 0.214286 | Acidobacteria | Blastocatellia (Subgroup 4) | | Blastocatellales | Blastocatellaceae | | *Blastocatella* | | | |
|  | OTU11292 | 2.788937 | 0 | Proteobacteria | Deltaproteobacteria | | Sva0485 | uncultured | |  | | | |
|  | OTU11264 | 2.697333 | 0.534063 | Proteobacteria | Gammaproteobacteria | | Gammaproteobacteria Incertae Sedis | Unknown Family | | uncultured | | | |
|  | OTU10011 | 2.625537 | 0.277778 | Bacteroidetes | Bacteroidia | | Cytophagales | Cyclobacteriaceae | | uncultured | | | |
| connector | OTU574 | 0 | 0.64 | Proteobacteria | Alphaproteobacteria | | Sphingomonadales | Sphingomonadaceae | | *Altererythrobacter* | | | |
|  | OTU12252 | -0.40078 | 0.64 | Acidobacteria | Acidobacteriia | | Solibacterales | Solibacteraceae (Subgroup 3) | | PAUC26f | | | |
|  | OTU12167 | -0.70711 | 0.625 | Proteobacteria | Gammaproteobacteria | | Cellvibrionales | Halieaceae | | *Halioglobus* | | | |
|  | OTU9289 | 2.392101 | 0.468783 | Epsilonbacteraeota | Campylobacteria | | Campylobacterales | Sulfurovaceae | | *Sulfurovum* | | | |
| CK | | | | | | | | | | | | |  |
| module hub | OTU888 | 3.199213 | 0 | Acidobacteria | Subgroup 21 | | uncultured | | |  | | | |
|  | OTU2803 | 3.522944 | 0 | Gemmatimonadetes | BD2-11 terrestrial group | | uncultured | | |  | | | |
|  | OTU3259 | 3.831169 | 0 | Proteobacteria | Deltaproteobacteria | | Desulfobacterales | Desulfobacteraceae | | | | | |
|  | OTU4628 | 3.583512 | 0 | Nitrospirae | Thermodesulfovibrionia | | uncultured | uncultured | |  | | | |
|  | OTU5072 | 3.296097 | 0 | Acidobacteria | Subgroup 22 | | uncultured | | |  | | | |
|  | OTU7209 | 2.850434 | 0.079861 | Proteobacteria | Gammaproteobacteria | | BD7-8 | uncultured | |  | | | |
|  | OTU9559 | 2.617538 | 0 | Acidobacteria | Subgroup 21 | | uncultured | | |  | | | |
|  | OTU10007 | 2.947822 | 0 | Proteobacteria | Gammaproteobacteria | | PLTA13 | uncultured | |  | | | |
|  | OTU14182 | 2.682307 | 0.083176 | Proteobacteria | Gammaproteobacteria | | Gammaproteobacteria Incertae Sedis | Unknown | |  | | | |
|  | OTU14200 | 2.806428 | 0 | Chloroflexi | Anaerolineae | | Anaerolineales | Anaerolineaceae | | | | | |

**Table S4 Keystone taxa of the SparCC networks under different treatments. The keystone taxa also identified in the time-series phylogenetic molecular ecological networks were marked in red.**

| **Roles** | **ID** | **Zi** | **Pi** | **Phylum** | **Class** | **Order** | **Family** | **Genus** | |
| --- | --- | --- | --- | --- | --- | --- | --- | --- | --- |
| BN | | | | | | | | |  |
| module hub | OTU13057 | 2.744 | 0.500 | Proteobacteria | Gammaproteobacteria | Ectothiorhodospirales | Thioalkalispiraceae | *Thioalkalispira* | |
|  | OTU4442 | 2.640 | 0.492 | Proteobacteria | Gammaproteobacteria | 1013-28-CG33 | uncultured |  | |
|  | OTU14669 | 4.775 | 0.000 | Proteobacteria | Gammaproteobacteria | B2M28 | uncultured |  | |
|  | OTU888 | 2.573 | 0.000 | Acidobacteria | Subgroup 21 | uncultured |  |  | |
|  | OTU5228 | 2.573 | 0.000 | Proteobacteria | Deltaproteobacteria | NB1-j | uncultured |  | |
|  | OTU5792 | 2.421 | 0.487 | Epsilonbacteraeota | Campylobacteria | Campylobacterales | Thiovulaceae | *Sulfurimonas* | |
| connector | OTU6142 | 0.439 | 0.631 | Epsilonbacteraeota | Campylobacteria | Campylobacterales | Sulfurovaceae | *Sulfurovum* | |
|  | OTU840 | -0.799 | 0.640 | Proteobacteria | Gammaproteobacteria | Cellvibrionales | Halieaceae | *Halioglobus* | |
|  | OTU3584 | -0.865 | 0.625 | Gemmatimonadetes | PAUC43f marine benthic group | uncultured bacterium | |  | |
| N | | | | | | | | |  |
| module hub | OTU13057 | 2.581 | 0.127 | Proteobacteria | Gammaproteobacteria | Ectothiorhodospirales | Thioalkalispiraceae | *Thioalkalispira* | |
|  | OTU5792 | 2.565 | 0.303 | Epsilonbacteraeota | Campylobacteria | Campylobacterales | Thiovulaceae | *Sulfurimonas* | |
|  | OTU12381 | 2.516 | 0.499 | Epsilonbacteraeota | Campylobacteria | Campylobacterales | Thiovulaceae | *Sulfurimonas* | |
|  | OTU405 | 3.945 | 0.000 | Proteobacteria | Gammaproteobacteria | Betaproteobacteriales | Nitrosomonadaceae | MND1 | |
| connector | OTU3780 | 1.361 | 0.653 | Proteobacteria | Gammaproteobacteria | Betaproteobacteriales | Hydrogenophilaceae | *Thiobacillus* | |
|  | OTU11201 | 0.059 | 0.628 | Acidobacteria | Thermoanaerobaculia | Thermoanaerobaculales | Thermoanaerobaculaceae | Subgroup 23 | |
|  | OTU1614 | 0.010 | 0.661 | Proteobacteria | Gammaproteobacteria | Acidiferrobacterales | Acidiferrobacteraceae | *Sulfurifustis* | |
|  | OTU9385 | -0.445 | 0.656 | Acidobacteria | Subgroup 18 | uncultured |  |  | |
|  | OTU15135 | -0.820 | 0.625 | Proteobacteria | Gammaproteobacteria | Cellvibrionales | Halieaceae | OM60(NOR5) clade | |
|  | OTU11825 | -0.885 | 0.667 | Chloroflexi | OLB14 | uncultured |  |  | |
|  | OTU438 | -0.901 | 0.667 | Chloroflexi | Anaerolineae | Anaerolineales | Anaerolineaceae | uncultured | |
|  | OTU6585 | -0.901 | 0.667 | Proteobacteria | Deltaproteobacteria | Myxococcales | BIrii41 | uncultured | |
| B | | | | | | | | |  |
| module hub | OTU778 | 2.789 | 0.317 | Bacteroidetes | Bacteroidia | Flavobacteriales | Flavobacteriaceae | *Robiginitalea* | |
|  | OTU5484 | 2.553 | 0.292 | Proteobacteria | Alphaproteobacteria | Sphingomonadales | Sphingomonadaceae | *Novosphingobium* | |
|  | OTU888 | 3.104 | 0.000 | Acidobacteria | Subgroup 21 | |  |  | |
|  | OTU3001 | 2.963 | 0.000 | Acidobacteria | Blastocatellia (Subgroup 4) | Blastocatellales | Blastocatellaceae | *Blastocatella* | |
|  | OTU3093 | 2.501 | 0.000 | Chloroflexi | Anaerolineae | Anaerolineales | Anaerolineaceae | uncultured | |
|  | OTU13779 | 2.944 | 0.000 | Proteobacteria | Gammaproteobacteria | Tenderiales | Tenderiaceae | *Candidatus* Tenderia | |
|  | OTU2583 | 2.632 | 0.000 | Chloroflexi | Anaerolineae | SBR1031 | uncultured |  | |
|  | OTU9289 | 2.475 | 0.236 | Epsilonbacteraeota | Campylobacteria | Campylobacterales | Sulfurovaceae | *Sulfurovum* | |
| CK | | | | | | | | |  |
| module hub | OTU13779 | 2.616 | 0.000 | Proteobacteria | Gammaproteobacteria | Tenderiales | Tenderiaceae | *Candidatus* Tenderia | |
|  | OTU13057 | 2.917 | 0.000 | Proteobacteria | Gammaproteobacteria | Ectothiorhodospirales | Thioalkalispiraceae | *Thioalkalispira* | |
|  | OTU4628 | 3.520 | 0.000 | Nitrospirae | Thermodesulfovibrionia | uncultured |  |  | |
|  | OTU14681 | 2.546 | 0.000 | Chloroflexi | Anaerolineae | SBR1031 | uncultured |  | |
|  | OTU7209 | 2.797 | 0.000 | Proteobacteria | Gammaproteobacteria | BD7-8 | uncultured |  | |
|  | OTU14182 | 2.797 | 0.000 | Proteobacteria | Gammaproteobacteria | Gammaproteobacteria Incertae Sedis | Unknown |  | |
|  | OTU10007 | 3.409 | 0.000 | Proteobacteria | Gammaproteobacteria | PLTA13 | uncultured |  | |
| connector | OTU888 | 2.601 | 0.547 | Acidobacteria | Subgroup 21 | uncultured |  |  | |
|  | OTU5533 | 0.658 | 0.622 | Acidobacteria | Subgroup 6 | uncultured |  |  | |
|  | OTU4110 | 1.541 | 0.667 | Chloroflexi | Dehalococcoidia | FS117-23B-02 | uncultured |  | |
|  | OTU14200 | 1.585 | 0.719 | Chloroflexi | Anaerolineae | Anaerolineales | Anaerolineaceae | uncultured | |

**Table S5 General genomic features of genomes in this study relative to *Sulfurimonas* and *Sulfurovum***

| **Genome** | **genome size** | **Complete-ness** | **expected genome size** | **No. of contigs/N50** | **No. of CDS** | **Ave. CDS size** | **% CDS regions** | **% GC** | **Genes** | **Homologs** | | **Homolog Families** | | **Singletons** |  |
| --- | --- | --- | --- | --- | --- | --- | --- | --- | --- | --- | --- | --- | --- | --- | --- |
| ***Sulfurovum*** | | | | | | | | | | | | | | | |
| ***Sulfurovum lithotrophicum*** | 2217891 | 98.9 | 2242559 | 1/2,217,891 | 2193 | 929 | 0.918574 | 44.3 | 2220 | | 1996 | | 1943 | 224 |  |
| ***Sulfurovum riftiae*** | 2374692 | 97.8 | 2428110 | 72/206,012 | 2368 | 911.9 | 0.90933 | 45.7 | 2455 | | 2122 | | 2049 | 333 |  |
| ***Sulfurovum* sp. 39-42-12** | 1840375 | 95.7 | 1923067 | 102/24,741 | 1855 | 914.3 | 0.921566 | 42.3 | 1909 | | 1853 | | 1843 | 56 |  |
| ***Sulfurovum* sp. 24-42-9** | 1948560 | 94.6 | 2059789 | 137/26,646 | 1949 | 903.5 | 0.903704 | 42 | 2072 | | 1953 | | 1919 | 119 |  |
| ***Sulfurovum* sp. 28-43-6** | 1349741 | 63 | 2142446 | 339/5247 | 1360 | 823.7 | 0.829961 | 43 | 1571 | | 1416 | | 1394 | 155 |  |
| ***Sulfurovum* sp. 35-42-20** | 1901697 | 97.8 | 1944475 | 162/19114 | 1906 | 908.3 | 0.910355 | 42.2 | 2006 | | 1940 | | 1911 | 66 |  |
| ***Sulfurovum* sp. 16-42-52** | 1987317 | 95.7 | 2076611 | 66/55,271 | 2024 | 904.6 | 0.921298 | 41.7 | 2070 | | 2009 | | 1991 | 61 |  |
| ***Sulfurovum* sp. 17-42-90** | 1964549 | 93.5 | 2101122 | 92/41662 | 2009 | 892.2 | 0.912387 | 41.7 | 2083 | | 1991 | | 1963 | 92 |  |
| ***Sulfurovum* sp. 4484 65** | 1127112 | 41.3 | 2729085 | 124/9332 | 1162 | 835.2 | 0.861052 | 38 | 1293 | | 1083 | | 1066 | 210 |  |
| ***Sulfurovum* sp. UBA2215** | 1517457 | 73.9 | 2053392 | 10/204,756 | 1504 | 926.4 | 0.918185 | 40.5 | 1565 | | 1337 | | 1318 | 228 |  |
| ***Sulfurovum* sp. AR** | 2125769 | 100 | 2125769 | 11/307,537 | 2153 | 904.9 | 0.916492 | 39.2 | 2175 | | 1852 | | 1796 | 323 |  |
| ***Sulfurovum* sp. NBC37-1** | 2562277 | 100 | 2562277 | 1/2562277 | 2554 | 917.8 | 0.914835 | 43.9 | 2603 | | 2240 | | 2160 | 363 |  |
| ***Sulfurimonas*** | | | | | | | | | | | | | | | |
| ***Sulfurimonas* sp. CG1 02 36 14** | 2360463 | 95.7 | 2466524 | 135/23,266 | 2315 | 908.9 | 0.891394 | 36 | 2477 | | 2125 | | 2029 | 352 |  |
| ***Sulfurimonas* sp. BM502** | 2391647 | 96.7 | 2473265 | 45/90,685 | 2544 | 859.1 | 0.913826 | 36.7 | 2601 | | 2193 | | 2102 | 408 |  |
| ***Sulfurimonas* sp. ES** | 2080587 | 95.7 | 2174072 | 206/28,163 | 2105 | 909.4 | 0.920071 | 33 | 2134 | | 1538 | | 1283 | 596 |  |
| ***Sulfurimonas* sp. BM702** | 1891063 | 95.7 | 1976032 | 122/26,298 | 2001 | 841.6 | 0.890526 | 34.4 | 2131 | | 1799 | | 1720 | 332 |  |
| ***Sulfurimonas* sp. GWF2 37 8** | 1906285 | 91.3 | 2087935 | 101/25,793 | 1888 | 900.6 | 0.891961 | 36.7 | 2030 | | 1771 | | 1737 | 259 |  |
| ***Sulfurimonas* sp. RIFCSPLOWO2 12 36 12** | 1906285 | 97.8 | 1949167 | 69/48,143 | 2211 | 909.5 | 1.054881 | 36.2 | 2299 | | 2019 | | 1951 | 280 |  |
| ***Sulfurimonas* sp. UBA2011** | 1596058 | 100 | 1596058 | 15/174,873 | 1744 | 836.2 | 0.913709 | 34.2 | 1790 | | 1492 | | 1464 | 298 |  |
| ***Sulfurimonas* sp. RIFOXYD12 FULL 33 39** | 2342198 | 100 | 2342198 | 13/322,821 | 2320 | 952.3 | 0.943275 | 34.4 | 2372 | | 2309 | | 2281 | 63 |  |
| ***Sulfurimonas s*p. RIFOXYD2 FULL 34 21** | 2360463 | 100 | 2360463 | 15/311,526 | 2345 | 949.3 | 0.943081 | 34.5 | 2395 | | 2310 | | 2280 | 85 |  |
| ***Sulfurimonas autotrophica* DSM 16294** | 2153198 | 100 | 2153198 | 1/2,153,198 | 2161 | 937.5 | 0.940897 | 35.2 | 2176 | | 1932 | | 1896 | 244 |  |
| ***Sulfurimonas hongkongensis*** | 2302023 | 100 | 2302023 | 28/235,215 | 2255 | 945.2 | 0.925893 | 34.9 | 2271 | | 2066 | | 2021 | 205 |  |
| ***Sulfurimonas denitrificans* DSM 1251** | 2201561 | 100 | 2201561 | 1/2,201,561 | 2163 | 951.2 | 0.934539 | 34.5 | 2182 | | 2033 | | 1979 | 149 |  |
| ***Sulfurimonas gotlandica* GD1** | 2952682 | 98.9 | 2985523 | 1/2,952,682 | 2874 | 963.8 | 0.938117 | 33.6 | 2887 | | 2460 | | 2286 | 427 |  |
| ***Sulfurimonas* sp. PC08-66** | 2294002 | 98.9 | 2319517 | 20/256,422 | 2348 | 934.2 | 0.95619 | 46.9 | 2350 | | 1890 | | 1819 | 460 |  |

**Table S6** **The completeness and contamination of *Sulfurimonas* and *Sulfurovum* MAGs**

|  | Sample | Bin ID | Completeness | Contamination |
| --- | --- | --- | --- | --- |
| *Sulfurovum* | BN60 | Bin007 | 70.33 | 14.66 |
|  | B60 | Bin033 | 71.68 | 8.95 |
|  | CK1 | Bin001 | 81.518 | 7.172 |
|  | N60 | Bin015 | 73.33 | 10.93 |
| *Sulfurimonas* | N60 | Bin055 | 81.33 | 4.29 |
|  | BN60 | Bin029 | 51.16 | 1.724 |

**Table S7** **The 16S rRNA gene sequences generated by hiTAIL PCR and their closest phylogenetic affiliation obtained using Blast**

| ID | Sequence length | Closest phylogenetic affiliation | Max Score | Total Score | Query Coverage | E value | Identity |
| --- | --- | --- | --- | --- | --- | --- | --- |
| H06 | 1105 | *Novosphingobium pentaromativorans* US6-1 | 1853 | 5033 | 95% | 0 | 98.05% |
| A07 | 1055 | *Novosphingobium pentaromativorans* US6-1 | 1872 | 5052 | 96% | 0 | 99.02% |
| B07 | 851 | *Novosphingobium pentaromativorans* US6-1 | 1504 | 4512 | 95% | 0 | 100.00% |
| C12 | 930 | *Novosphingobium pentaromativorans* US6-1 | 1578 | 4734 | 92% | 0 | 99.88% |
| D12 | 1017 | *Novosphingobium pentaromativorans* RC2 | 1779 | 1779 | 95% | 0 | 99.79% |
| B03 | 1187 | *Novosphingobium pentaromativorans* US6-1 | 420 | 420 | 66% | 2.00E-112 | 76.57% |
| C03 | 1167 | *Novosphingobium pentaromativorans* US6-1 | 1832 | 5342 | 94% | 0 | 96.76% |
| D03 | 1251 | *Novosphingobium pentaromativorans* US6-1 | 2015 | 2015 | 97% | 0 | 96.80% |
| E03 | 1228 | *Novosphingobium pentaromativorans* US6-1 | 1925 | 5513 | 97% | 0 | 95.57% |
| F03 | 1148 | *Novosphingobium pentaromativorans* US6-1 | 1929 | 1929 | 97% | 0 | 97.54% |
| G03 | 1158 | *Novosphingobium pentaromativorans* US6-1 | 1888 | 1888 | 96% | 0 | 97.24% |
